# Effects of AZFc (b2/b4, b1/b3, b2/b3, and gr/gr) deletions and primary duplications on the outcomes of the first intracytoplasmic sperm injection treatment cycle: A single‐center retrospective cohort study

**DOI:** 10.1111/andr.13818

**Published:** 2024-12-13

**Authors:** Linlin Li, Xiangyin Liu, Xinying Wang, Hongguo Zhang, Ruizhi Liu

**Affiliations:** ^1^ Reproductive Medicine Center, Prenatal Diagnosis Center First Hospital of Jilin University Changchun China

**Keywords:** cumulative reproductive outcomes, intracytoplasmic sperm injection, next‐generation sequencing, partial AZFc deletion, primary AZFc duplication

## Abstract

**Background:**

Current advances in high‐throughput sequencing technology enable the precise identification of Y chromosome microdeletion and primary duplication in infertile couples, but the mechanism and clinical significance of these mutations in assisted reproductive techniques remain unclear.

**Objectives:**

To investigate the effects of AZFc (b2/b4, b1/b3, b2/b3, and gr/gr) deletions and primary duplications on the outcomes of the first intracytoplasmic sperm injection (ICSI) treatment cycle.

**Methods:**

Y chromosome microdeletions and primary duplications in infertile men were detected using next‐generation sequencing (NGS) technology. A total of 813 patients undergoing their first ICSI treatment were divided into six groups: b2/b4 deletion group (*n* = 28), three partial AZFc subdeletion groups (b1/b3, *n* = 13; b2/b3, *n* = 72; gr/gr, *n* = 71), primary AZFc duplication group (*n* = 54), and control group with a normal Y chromosome (*n* = 575). The multivariate logistic regression analyses were conducted to assess and compare the embryologic and cumulative reproductive outcomes of ICSI treatment across these groups.

**Results:**

Compared with the control group, the b2/b4 deletion group showed a poor ICSI embryologic outcome after ICSI treatment, with a significantly lower fertilization rate per oocytes retrieval (72.22% vs.79.89%; adjusted odds ratio [OR], 0.63; 95% confidence interval [CI], 0.45–0.88, *p* < 0.01) and 2 pronuclear (2PN) fertilization rate (66.37% vs. 74.80%; adjusted OR, 0.65; 95% CI, 0.47–0.89, *p* < 0.01) either before or after adjustment for confounding factors. Nevertheless, three partial AZFc deletion groups showed no effect on the ICSI fertilization rate after ICSI treatment. The primary AZFc duplication group had a significantly lower clinical pregnancy rate per transferred embryo (56.25% vs. 65.97%; adjusted OR, 0.64; 95% CI, 0.41–0.99, *p* < 0.05), and the semen characteristics varied from azoospermia to normozoospermia. In addition, all indicators related to embryo quality, clinical pregnancy, and live birth outcomes in the primary duplication group were inferior to those in the control group.

**Conclusion:**

This study indicates that b2/b4 deletion has a negative effect on ICSI outcomes, particularly the fertilization rates. Partial AZFc deletions have no significant effect on the fertilization rate after ICSI treatment. Primary AZFc duplication can lead to varying seminal phenotypes and has a negative effect on ICSI embryologic and pregnancy outcomes, particularly showing a significant association with low birth weight in newborns after ICSI treatment.

## 1 INTRODUCTION

Azoospermia factor (AZF; OMIM 415000) in the male‐specific region of the Y‐chromosome (MSY) is an essential genomic segment for spermatogenesis.^1^ Located on the long arm of the Y chromosome, AZF regions have three subregions referred to as AZFa, AZFb, and AZFc, AZFc deletion (60–70%) is most frequent in infertile men affected by AZF microdeletions, followed by AZFa (0.5–4%), AZFb (1–5%), and AZFb+c (1–3%) deletions.^2^ The AZFc with palindromic structures and repetitive sequences is more susceptible to genomic rearrangements mediated by nonallelic homologous recombination (NAHR) during meiosis, thus causing a significant copy number variation (CNV) in the AZFc region, and the deletion and duplication rearrangements are very common in this region.^3^ It is thought that complete AZFc deletions are thought to be associated with severe oligozoospermia, and even azoospermia.^4^ However, the association of partial AZFc deletions with male infertility is not clearly established as in the case of complete AZFc deletions, and there is wide heterogeneity in the phenotypes and clinical symptoms of patients with partial AZFc deletions.^5^, ^6^ Meanwhile, AZF duplications have long been neglected because of technical limitations of the sequence‐tagged site (STS)‐based approach, and the recent advancements in research have shed new light on this area. A state of art research has described the associations between primary AZFc duplications and male infertility in specific Y‐haplogroups.^1^ However, overall, there are still few studies on the association between primary AZFc duplications and male infertility. This recent finding underscores the importance of further investigation into AZF duplications and their potential effects on male fertility across different genetic backgrounds. It was reported that partial AZFc deletions are associated with an increased risk of complete AZFc deletion.^7^ The genomic duplications in AZFc and the instability of the Y chromosome might be associated with spermatogenesis.^8^ Furthermore, the research conducted by Yang et al.^9^ suggested that the AZFc‐mutated structures with excessive NAHR‐substrate exert a significantly negative effect on spermatogenesis. In addition, Concurrent partial genomic deletion and duplications in the AZFc region can impair the spermatogenic function more severely than partial genomic deletion in the AZFc region alone.

Next‐generation sequencing (NGS) technology and analysis methods have rapidly evolved and are increasingly being used in research and clinical settings.^10^ Compared with the traditional two‐step multiplex PCR method in guidelines for detecting CNV in the Y chromosome C region, NGS technology has the advantages of high throughput, high sequencing volume, accurate data acquisition, good sequence coverage, and depths, the ability to detect minor variants such as deletions, duplications, and levels of mosaicism. In addition, it does not require electrophoretic separation of sequencing products. Meanwhile, the NGS technology has its own disadvantages, such as high cost, complex data analysis, and the absence of standardized protocols.^11^ To address these challenges, we have developed a unique capture sequencing method, which utilizes specific STSs on the Y chromosome to accurately determine the full spectrum of deletions and duplications, and identify specific Y chromosomal structures. However, except for b2/b4 deletion, the clinical relevance of gene mutations in the evaluation of infertile couples, particularly those undergoing assisted reproductive techniques (ART), remains uncertain. Several studies^12^, ^13^, ^14^, ^15^, ^16^, ^17^ have investigated the relationship between AZFc complete deletion (b2/b4 deletion) and clinical outcomes after ICSI treatment, but the findings are controversial. Previous researches^14^, ^18^ have consistently shown a lower fertilization rate in individuals with AZFc deletions compared with those with a normal Y chromosome, this is likely because of poor semen quality. Nevertheless, as described above, partial deletions in the AZFc region produce a wide range of seminal phenotypes from normozoospermia to oligozoospermia or azoospermia. To date, the effects of these mutations, specifically partial AZFc deletions or duplications, on ICSI fertilization and pregnancy outcomes remain unknown. This information is crucial for clinicians involved in genetic counseling.

In this retrospective cohort study, an NGS read‐depth approach was used to detect AZF rearrangements in infertile couples undergoing ICSI‐assisted reproduction. The patients with AZFc deletions or duplications were divided into five groups: complete AZFc deletion group (b2/b4 deletion), three partial AZFc deletion groups (b1/b3, b2/b3, and gr/gr deletions with or without secondary duplication), and primary AZFc duplication group (with duplication only, no deletion). We investigated the effects of AZFc deletions and duplications on the clinical outcomes of patients undergoing ICSI‐embryo transfer in comparison to control patients with a normal Y chromosome. The patients with complete AZFc deletion had poor outcomes after ICSI treatment, particularly in the fertilization rate and 2 pronuclear (2PN) fertilization rate. Conversely, partial AZFc deletions did not exert significant effects on the fertilization rate after ICSI treatment. The presence of primary AZFc duplications resulted in various seminal phenotypes varying from azoospermia to normozoospermia and had a detrimental effect on ICSI embryologic and overall pregnant outcomes, particularly showing a significant association with low birth weight in newborns after ICSI treatment.

## 2 MATERIALS AND METHODS

### 2.1 Study population

A retrospective study was carried out in infertile couples with or without AZFc deletion or duplication undergoing ICSI treatments for a period of 5 years from June 2017 to December 2022 in the Center of Reproductive Medicine, First Hospital of Jilin University, Changchun, China, and they were followed up for 1–6 years until December 2023. Before ICSI treatment, the Y chromosome microdeletion testing, based on NGS methods, is conducted as part of the routine pre‐ICSI examination for male infertility patients. All patients with CNVs in the AZF region included in this study were well informed that potential deletions or duplications in the AZFc region could be transmitted to their offspring, which may result in impaired sperm production in male offspring.

Patient inclusion and exclusion criteria were as follows: the infertile couples undergoing the first fresh ICSI cycle and all following freeze–thaw cycles during the same ovarian stimulation cycle were included in this study. The infertile couples undergoing non‐first ICSI cycles were excluded. In order to avoid possible confounding factors affecting the study results, the infertile couples with abnormal abnormality in both partners, female partners ≥35 years old or poor ovarian responder (number of oocytes retrieved <5), those in the frozen‐thawed oocyte cycle and oocyte/sperm donation cycles or undergoing early‐rescue ICSI or late‐rescue ICSI, and those without final pregnancy outcomes were excluded from this study. In addition, the infertile couples with male partners who had partial AZFa and AZFb variations were also excluded from this study. The patients were divided into six groups according to specific deletions and duplications such as b2/b4 deletion, three partial AZFc deletions with or without secondary duplication (b1/b3, b2/b3, gr/gr deletions), primary AZFc duplications without deletion, and a control group with a normal Y chromosome.

### 2.2 Ethical approval

This study was approved by the ethics committee of the First Hospital of Jilin University (approval no.: 2021–741). Informed consent was obtained from all of the study subjects before treatment.

### 2.3 AZF copy number variation analysis

The screening for Yq microdeletions was performed using both the target area probe capture technology and the NGS technology on the MiSeqDx platform (Illumina, Inc.). This approach was designed to identify CNV in specific regions of the Y chromosome in infertile men with both genomic deletions and duplications. The capture probes were designed based on the genomic sequence information of the AZF region on the Y chromosome obtained from the human genome database (http://genome.ucsc.edu/). A three‐segment probe was used in this study. The main principles for probe design were as follows: (1) specificity: three‐segment probe must be unique in the human genome, with a total of 60 bp in length, and 20 bp for each segment. However, because of repetitive and palindromic sequences in the AZFb and AZFc regions, it was impractical to ensure the uniqueness of the probe design. Therefore, it was necessary to ensure that this probe could only be paired with complementary base in the target AZF region, with a specified copy number, and it could not be paired with complementary base in other regions; (2) complementarity: the probes were engineered to bind exclusively to the fully complementary sequences in the target region; (3) internal structure: the probes must be devoid of self‐complementary sequences to prevent secondary structure formation; (4) guanine–cytosine (GC) content: when the probe is designed, it is important to maintain a GC content of approximately 50%, as this helps to ensure a consistent annealing temperature. Given the complex repetitive and palindromic structures present in the AZFb and AZFc regions, both the base sequence information and structural characteristics of the AZF regions should be considered comprehensively during the probe design (Figure S1). The probes should be evenly distributed across the AZF region to ensure comprehensive coverage. After screening, a total of 206 sets of oligonucleotide probes were designed to facilitate the precise identification of the AZF region on the Y chromosome (Figure S2). Of the above 206 probe loci, 165 were located within the AZF region, 10 on the short arm of the Y chromosome, 9 on the X chromosome, and 22 on the autosomes were retained after screening. The autosomal loci served as internal references to analyze the detected signal values of other loci. The loci on the short arm of the Y chromosome were used in combination with the loci on X chromosome to analyze the sex chromosome composition in the samples. Additionally, when the Y chromosome copy number was not unique, these loci on the short arm of the Y chromosome were used in combination with autosomal loci to analyze abnormalities in the AZF region.

NGS analysis was performed using a Y chromosome microdeletion detection kit (Jabrehoo) according to the manufacturer's protocols. The main steps included DNA extraction, library preparation, target enrichment sequencing, and data analysis. The experimental procedures were conducted with reference to the methods used in our previously published literature.^19^, ^20^ After sequencing, a software independently developed by Jabrehoo Med Tech Co., Ltd. was used to identify the microdeletions in the AZF region of the Y chromosome. First, the reads were processed and filtered using Trimmomatic software to remove adapter sequences and low‐quality sequences.^21^ Then the processed reads were aligned to the human reference genome (hg19) using BWA software,^22^ and the number of sequences corresponding to each amplicon‐specific probe was quantified to determine the value of initial signals for each amplicon region. Given that the reference region was established based on the autosomal region, a standard value for single‐copy sequence signal detection was set as half the initial signal detection value derived from the reference region. After that, the detection value of initial signals derived from each amplicon region was normalized based on the detection value of the standard single‐copy sequence signals to obtain the final signal detection values. Thereafter, the test samples and typical abnormal samples were projected into a multidimensional space with each amplicon as an axis. The most likely typical CNVs in the AZF region in test samples were assessed using the Euclidean geometric distance method, and the initial copy number of each amplicon was determined. Finally, the detection values of signals derived from each amplicon were validated based on the preliminary assessment values using the Z‐score method, thus further confirming the analysis results (Figure S3).

### 2.4 Real‐time quantitative PCR validation

We used real‐time quantitative PCR (qPCR) to validate and confirm the various deletions and duplications in the AZF region. Comprehensive details regarding primer sequence designs, their precise localization in the AZF region, and corresponding anticipated results are provided in Figure S4 and Tables S1 and S2. Genomic DNAs were extracted from 200 µL blood samples using QIAamp DNA Blood Mini Kit (Qiagen). qPCR assays were performed using Hieff UNICON qPCR SYBR Green Master Mix (No Rox) (YEASEN Biotech Co., Ltd.; cat. no. 11201ES03). A 20 µL reaction mixture, which contained 10 µL SYBR Green Master Mix, 50 ng template DNA (approximately 1–2 µL, a volume calculated according to the corresponding concentrations), and 0.75 µL each of the forward and reverse primers (10 µM stock), was prepared, and then the nuclease‐free water was used to adjust the final volume. Real‐time PCR amplification was conducted using a real‐time PCR system under the following thermal cycling conditions: initial denaturation at 95°C for 5 min, followed by 40 cycles of denaturation at 95°C for 10 s, annealing at 60°C for 20 s, and extension at 72°C for 60 s. Postamplification melting‐curve analysis was performed using the default settings. All samples were analyzed in triplicate. The male and female samples with negative NGS detection results were selected as the control group. ZFXY was employed as a reference gene. Data analysis was performed by StepOne Software using the ΔΔCT method. The qPCR validation results for various deletions and duplications in the AZFc region, along with their corresponding NGS results, are presented in Figures S5 and S6.

### 2.5 ICSI protocols and laboratory procedures

Ovarian stimulation was performed using the following protocols: gonadotropin‐releasing hormone (GnRH) antagonist protocol (77.37%), GnRH agonist protocol (19.80%), or mild stimulation protocol (2.83%). Detailed ovarian stimulation, triggering, oocyte retrieval, and laboratory procedures were performed referring to the previous study.^23^ When one dominant follicle reached 20 mm in diameter, three follicles reached 18 mm in diameter, or four follicles reached 16 mm, hCG (Lishenbao, Livzon Pharmaceutical Co., Ltd.) 3000–10,000 IU, Ovidrel (Merck Serono S.p.A) 250 µg or triptorelin (Tiantai Mountain Pharmaceutical Company) 0.1 mg was intramuscularly injected selectively to trigger final maturation of the oocytes. Oocyte retrieval took place 36–40 h after triggering. ICSI was performed by using a pipette injection under micromanipulator control on the day of oocyte retrieval (day 0). A good‐quality embryo on day 3 was defined as 6–10 cell embryos, with a fragmentation rate of 20% or less, and intact blastomeres, without multinucleation and vacuolation. The expanded blastocysts on days 5 and 6 were counted using the Gardner grading system.^24^

### 2.6 Embryo transfer and pregnancy outcomes

Embryo transfer was performed either on day 3 (cleavage stage) or day 5 (blastocyst stage) in the fresh embryo transfer cycle and frozen–thawed embryo transfer cycle. All patients were given luteal phase support starting on the day of embryo transfer (Chrinone, Merck). The embryologic outcomes included the fertilization rate, 2PN cleavage rate, high‐quality embryo rate, and blastocyst formation rate. The reproductive outcomes included positive hCG at 2 weeks after embryo transfer, ongoing pregnancy at gestational weeks 7–12, and live birth at delivery. Positive hCG was defined as plasma hCG > 5.3 IU/L. Clinical pregnancy was defined as the presence of an intrauterine gestational sac with or without a fetal heartbeat on ultrasonography during the first trimester. The ongoing pregnancy was defined as a clinical pregnancy that continued for at least 12 weeks. The live birth was defined as the birth of at least a living child regardless of the duration of gestation. The miscarriage rate was defined as the fact that the total number of miscarriages before delivering a healthy fetus was divided by the number of total cycles of women with clinical pregnancy. The time to live birth refers to the time from the ovarian stimulation to a live birth.

### 2.7 Statistical methods

The statistical analysis was performed using Empower Stats software(X&Y Solutions, Inc) and R software version 4.1(R Foundation for Statistical Computing). The normally distributed continuous variables were presented as mean ± standard deviation (mean ± SD), and the normally distributed continuous variables were presented as median + interquartile range. Different continuous variables in various groups were compared using a one‐way ANOVA test or Kruskal–Wallis rank sum tests; the categorical variables were presented as counts and proportions and were compared with the baseline data using Pearson χ^2^ or Fisher's exact test. A two‐tailed *p*‐value < 0.05 was considered statistically significant.

In a generalized additive model, multivariate logistic regression analyses were conducted to confirm and compare the differences and similarities between the five AZFc deletion or duplication groups (b2/b4 deletion, b1/b3 deletion, b2/b3 deletion, gr/gr deletion, primary duplications) and the control group. The outcome indicators were previously described^14^ and adjusted for various factors such as female age, female body mass index (BMI), duration of infertility, female anti‐Mullerian hormone (AMH), basal antral follicle count (bAFC), metaphase II (MII) oocytes, male age, male BMI, seminal concentration, semen type, sperm extraction method, number of transferred cycles, and number of embryos transferred. An adjusted model was then established to estimate the odds ratio (OR) and 95% confidence interval (CI) for all outcomes in ICSI treatment.

## 3 RESULTS

### 3.1 Clinical characteristics of patients

**FIGURE 1:**
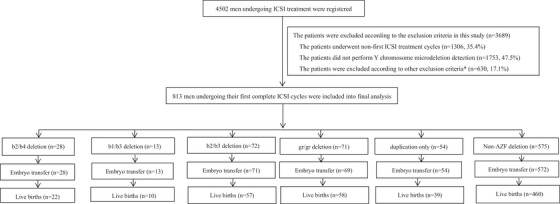
Flowchart of patient selection and outcomes. Note: *Other exclusion criteria include infertile couples with abnormal karyotype (*n* = 31), female age ≥35 years (*n* = 378), or oocytes retrieved <5 (*n* = 104), those undergoing oocyte freezing cycles (*n* = 2), or sperm donation cycles (*n* = 1), those without final pregnancy outcomes (*n* = 105), those with other types of AZF microdeletions besides AZFc deletion (*n* = 9).

A total of 813 patients undergoing ICSI treatment were included in this study, who were further assigned into six groups: five AZFc deletion or duplication (exposed) groups (*n* = 238), and the control (unexposed) group (*n* = 575). In five AZFc deletion or duplication groups, 28 patients had b2/b4 deletion, 13 had b1/b3 deletion, 72 had b2/b3 deletion, 71 had gr/gr deletion, and 54 had primary AZFc duplications. The inclusion and exclusion criteria are listed in detail in Figure 1. The proportion of AZFc deletion or duplication type and mode are exhibited in Table 1. The distribution of AZFc rearrangements, including partial deletion, duplication, and their combination varied among three partial AZFc deletion groups (b1/b3, b2/b3, gr/gr deletions). The prevalence of complex CNV (deletion + duplication) was highest in the b1/b3 deletion group (69.23%), followed by the b2/b3 deletion group (41.67%) and the gr/gr deletion group (14.08%).

**TABLE 1:** Percentages of various types of AZFc deletion or duplication.

Type	Mode	*n* (%)
b2/b4 del	b2/b4 del	28 (100%)
b1/b3 del^a^	b1/b3 del‐only	4 (30.77%)
	b2/b4 dup+b1/b3 del	1 (7.69%)
	gr/gr dup+b1/b3 del	8 (61.54%)
	Total	13
b2/b3 del	b2/b3 del‐only	42 (58.33%)
	b2/b3 del+b3/b4 dup	21 (29.17%)
	b2/b3 del+grey1,r1,r2 dup	3 (4.17%)
	b2/b3 del+grey1,r2 dup	6 (8.33%)
	Total	72
gr/gr del	gr/gr del‐only	61 (85.92%)
	b2/b3 del+b2/b4 dup	7 (9.86%)
	b2/b3 del+b2/b4 dup twice	3 (4.23%)
	Total	71
dup^b^ only	gr/gr dup	30 (55.56%)
	gr/gr dup twice	2 (3.70%)
	b2/b3 dup	17 (31.48%)
	b2/b4 dup	3 (5.56%)
	b1/b3 dup	1 (1.85%)
	t1/t2 dup	1 (1.85%)
	Total	54

^a^ Deletion.

^b^ Duplication.

The clinical characteristics of patients in each group are detailed in Table 2. No significant differences were observed among the six groups in terms of female indicators such as female age, duration of infertility, basal serum luteinizing hormone (LH) and AMH levels, and cause of infertility. It was observed that the bAFC was significantly lower in the control group compared with the b1/b3 deletion and b2/b3 deletion groups. In addition, there was a statistically significant difference in female BMI among the b2/b3 deletion, duplication, and control groups. In contrast, there were no statistically significant differences in male indicators such as age, BMI, seminal volume, semen type, and sperm extraction method across various groups. It was also noted that the b2/b4 deletion group exhibited the lowest testicular volume and seminal concentration among all groups, indicating a statistically significant difference when compared with the control group (*p* < 0.05). Furthermore, the cumulative reproductive outcomes such as the number of transferred cycles and the number of embryos transferred were monitored and compared among all groups, and the results indicated no statistically significant differences in the above two indicators.

**TABLE 2:** Clinical characteristics of infertile couples with and without AZFc deletion or duplication after undergoing ICSI treatment.

Clinical characteristic	Non‐AZF deletion (*n* = 575)	b2/b4 deletion (*n* = 28)	b1/b3 deletion (*n* = 13)	b2/b3 deletion (*n* = 72)	gr/gr deletion (*n* = 71)	duplication only (*n* = 54)
Female age (years)	29.66 ± 2.85	28.89 ± 2.57	28.62 ± 2.90	29.62 ± 3.03	29.14 ± 3.23	29.69 ± 3.16
Female BMI (kg/m^2^)	22.92 ± 3.62	22.46 ± 3.43	23.35 ± 3.89	23.81 ± 3.77*	22.60 ± 3.51	24.12 ± 4.04*
Duration of infertility (years)	3.00 (2.00–5.00)	3.00 (1.00–4.00)	3.00 (1.00–3.00)	3.50 (2.00–6.00)	3.00 (2.00–5.0)	3.50 (2.00–5.00)
Female basal LH (mU/L)	4.90 (3.61–6.70)	5.03 (4.05–6.75)	5.05 (3.06–7.40)	4.71 (3.84–6.33)	4.87 (3.71–6.5)	4.95 (3.25–6.40)
Female AMH (µg/L)	4.77 (2.98–7.32)	5.08 (3.45–6.64)	5.22 (3.75–6.29)	5.29 (3.62–8.38)	5.33 (3.49–7.2)	4.63 (2.79–6.34)
Basal antral follicle count	19.15 ± 5.80	20.79 ± 4.46	23.08 ± 8.68*	21.33 ± 5.65**	19.65 ± 7.15	20.67 ± 5.32
Male age (years)	31.65 ± 4.13	30.43 ± 3.05	30.46 ± 3.10	31.69 ± 4.23	30.99 ± 3.51	32.04 ± 4.83
Male BMI (kg/m^2^)	25.53 ± 4.05	25.44 ± 3.98	24.59 ± 3.12	25.86 ± 4.43	25.04 ± 3.61	26.32 ± 4.53
Testicular volume (mL)	11.81 ± 4.07	10.17 ± 3.89*	12.40 ± 3.51	12.46 ± 4.01	11.19 ± 3.29	12.06 ± 3.78
Cause of infertility, *n* (%)						
Male factor	418 (72.70%)	27 (96.43%)	10 (76.92%)	48 (66.67%)	63 (88.73%)	39 (72.22%)
Tubal	26 (4.52%)	0 (0.00%)	0 (0.00%)	8 (11.11%)	2 (2.82%)	3 (5.56%)
Endometriosis	2 (0.35%)	0 (0.00%)	0 (0.00%)	1 (1.39%)	0 (0.00%)	0 (0.00%)
Anovulatory	4 (0.70%)	0 (0.00%)	0 (0.00%)	1 (1.39%)	0 (0.00%)	0 (0.00%)
Unexplained	12 (2.09%)	0 (0.00%)	0 (0.00%)	2 (2.78%)	0 (0.00%)	1 (1.85%)
Mixed factors	105 (18.26%)	1 (3.57%)	3 (23.08%)	11 (15.28%)	6 (8.45%)	11 (20.37%)
Other causes	8 (1.39%)	0 (0.00%)	0 (0.00%)	1 (1.39%)	0 (0.00%)	0 (0.00%)
Seminal volume (mL)	3.42 ± 1.82	3.88 ± 1.39	3.68 ± 1.84	3.33 ± 1.61	3.66 ± 1.69	3.53 ± 1.70
Seminal concentration (10^6^/mL)	6.00 (0.10–28.50)	0.10 (0.00–0.75)***	19.40 (0.00–33.6)	10.60 (1.15–32.3)	10.90 (0.65–30.5)	15.40 (1.70–45.40)
No. of men with azoospermia, *n* (%)	151 (26.31%)	10 (35.71%)***	5 (38.46%)	12 (16.67%)	10 (14.29%)	5 (9.26%)
No. of men with severe oligozoospermia, *n* (%)	129 (22.47%)	16 (57.14%)***	0 (0.00%)	13 (18.06%)	22 (31.43%)	13 (24.07%)
No. of men with mild oligozoospermia, *n* (%)	91 (15.85%)	2 (7.14%)***	1 (7.69%)	17 (23.61%)	10 (14.29%)	9 (16.67%)
No. of men with normal sperm concentration, *n* (%)	203 (35.37%)	0 (0.00%)***	7 (53.85%)	30 (41.67%)	28 (40.00%)	27 (50.00%)
No. of transferred cycles	1.42 ± 0.73	1.43 ± 0.69	1.69 ± 0.75	1.33 ± 0.63	1.30 ± 0.60	1.48 ± 0.69
No. of embryos transferred	2.28 ± 1.26	2.18 ± 1.22	2.54 ± 1.13	2.12 ± 1.19	2.14 ± 1.05	2.48 ± 1.22
Semen type, *n* (%)						
Fresh, *n* (%)	485 (84.35%)	23 (82.14%)	13 (100.00%)	66 (91.67%)	65 (91.55%)	50 (92.59%)
Frozen, *n* (%)	90 (15.65%)	5 (17.86%)	0 (0.00%)	6 (8.33%)	6 (8.45%)	4 (7.41%)
Sperm extraction method, *n* (%)						
Ejaculated, *n* (%)	456 (79.30%)	24 (85.71%)	8 (61.54%)	60 (83.33%)	59 (83.10%)	48 (88.89%)
Testicular sperm, *n* (%)	119 (20.70%)	4 (14.29%)	5 (38.46%)	12 (16.67%)	12 (16.90%)	6 (11.11%)

*Note*: Non‐AZF deletion group serves as the reference. Data are presented as mean ± SD for normally distributed variables, median (IQR) for non‐normally distributed variables, and n (%) for categorical variables. Asterisks (*) indicate significant differences compared with the non‐AZF deletion group. Significance levels: *p ≤ 0.05, **p ≤ 0.01, ***p ≤ 0.001.

### 3.2 ICSI embryologic and cumulative reproductive outcomes

The embryologic and cumulative reproductive outcomes of ICSI treatment are detailed in Tables 3, 4, 5, 6. All participating infertile couples underwent their initial ICSI cycle, which encompassed the first fresh cycle and any subsequent freeze‐thaw cycles resulting from the same ovarian stimulation. The cumulative success rate per transferred embryo or per woman was calculated for various indicators. Comparative analyses of the embryologic and cumulative reproductive outcomes were conducted between the control group and other specified AZF deletion or duplication groups, respectively.

**TABLE 3:** Embryologic and cumulative reproductive outcomes of male patients with or without AZFc deletion or duplication after undergoing ICSI treatment.

Indicators	Non‐AZF deletion (*n* = 575)	b2/b4 deletion (*n* = 28)	b1/b3 deletion (*n* = 13)	b2/b3 deletion (*n* = 72)	gr/gr deletion (*n* = 71)	duplication only (*n* = 54)
Fertilization rate per oocyte retrieval, % (*n*)	79.89 (5459/6833)	72.22 (247/342)***	81.22 (160/197)	80.36 (798/993)	80.11(709/885)	77.63(472/608)
2PN fertilization rate, % (*n*)	74.80 (5111/6833)	66.37 (227/342)***	79.70 (157/197)	76.13 (756/993)	76.95(681/885)	74.51 (453/608)
2PN cleavage rate, % (*n*)	98.40 (5029/5111)	99.12 (225/227)	99.36 (156/157)	99.47 (752/756)*	97.36(663/681)***	98.01 (444/453)
High‐quality embryo rate, % (*n*)	52.53 (2685/5111)	46.70 (106/227)*	54.78 (86/157)	51.85 (392/756)	53.30(363/681)	51.88(235/453)
Blastocyst formation rate, % (*n*)	62.24 (2065/3318)	50.00 (77/154)***	53.27 (57/107)*	67.16 (362/539)*	61.62(281/456)	60.14 (175/291)
Implantation rate, % (*n*)	51.91 (680/1310)	44.26 (27/61)	63.64 (21/33)	52.94 (81/153)	59.21 (90/152)*	44.03 (59/134)*
Biochemical pregnancy rate per transfer, % (*n*)	72.48 (590/814)	72.50 (29/40)	72.73 (16/22)	77.08 (74/96)	76.09 (70/92)	65.00 (52/80)
Clinical pregnancy rate per transfer, % (*n*)	65.97 (537/814)	60.00 (24/40)	68.18 (15/22)	67.71 (65/96)	72.83 (67/92)	56.25(45/80)*
Ongoing pregnancy rate per transfer, % (*n*)	58.11 (473/814)	57.50 (23/40)	50.00 (11/22)	60.42 (58/96)	65.22 (60/92)	48.75(39/80)
Miscarriage rate per pregnancy, % (*n*)	13.04 (70/537)	8.33 (2/24)	33.33 (5/15)*	10.77 (7/65)	10.45(7/67)	13.33(6/45)
Live birth rate per transfer, % (*n*)	57.00 (464/814)	55.00 (22/40)	45.45 (10/22)	60.42 (58/96)	64.13 (59/92)	48.75 (39/80)
Biochemical pregnancy rate per woman, % (*n*)	90.43 (520/575)	89.29 (25/28)	92.31 (12/13)	87.50 (63/72)	85.92(61/71)	87.04 (47/54)
Clinical pregnancy rate per woman, % (*n*)	86.78(499/575)	85.71 (24/28)	84.62 (11/13)	83.33 (60/72)	85.92(61/71)	79.63(43/54)
Ongoing pregnancy rate per woman, % (*n*)	80.35 (462/575)	82.14 (23/28)	84.62 (11/13)	79.17 (57/72)	83.10(59/71)	72.22(39/54)
Live birth rate per woman, % (*n*)	80.00 (460/575)	78.57 (22/28)	76.92 (10/13)	79.17 (57/72)	81.69(58/71)	72.22(39/54)
No embryo suitable for transfer cycles rate, % (*n*)	0.52 (3/575)	0 (0/28)	0 (0/13)	1.39 (1/72)	2.82(2/71)*	0(0/54)

*Note*: Non‐AZF deletion group serves as the reference. Data are presented as % (*n*/*N*) for all variables. Asterisks (*) indicate significant differences compared with the non‐AZF deletion group. Significance levels: *p ≤ 0.05, **p ≤ 0.01, ***p ≤ 0.001.

**TABLE 4:** The parameters and outcomes of newborns from a complete ICSI cycle including fresh and frozen–thawed embryo transfer for male patients with or without AZFc deletion or duplication.

Overall outcome	Non‐AZF deletion (*n* = 575)	b2/b4 deletion (*n* = 28)	b1/b3 deletion (*n* = 13)	b2/b3 deletion (*n* = 72)	gr/gr deletion (*n* = 71)	Duplication only (*n* = 54)
Time to pregnancy from the ovarian stimulation (months)						
Time to first positive hCG, median	2.0 (1.0–3.0)	2.0 (1.0–3.0)	3.0 (2.0–4.0)	2.0 (1.0–3.0)	2.0 (1.0–3.0)	2.0 (1.0–3.5)
Time to first clinical pregnancy, median	3.0 (2.0–4.0)	3.0 (1.0–5.0)	3.0 (3.0–4.5)	3.0 (2.0–4.0)	3.0 (2.0–4.0)	3.0 (2.0–4.0)
Time to first ongoing pregnancy, median	5.0 (4.0–6.0)	5.0 (3.5–7.0)	7.0 (5.0–12.5)	5.0 (4.0–6.0)	5.0 (4.0–7.0)	5.0 (4.0–6.0)
Time to first live birth median, median	10.0(9.0–12.0)	10.0 (9.0–11.8)	12.5(11.0–18.0)	10.0 (9.0–12.0)	11.0 (9.0–13.0)	10.0 (9.0–11.5)
Newborn parameters						
Singleton live birth delivery rate, % (*n*)	76.09 (350/460)	95.45 (21/22)*	70.00 (7/10)	77.19 (44/57)	70.69 (41/58)	66.67 (26/39)
Twin live birth delivery rate, % (*n*)	23.91 (110/460)	4.55 (1/22)	30.00 (3/10)	22.81 (13/57)	29.31 (17/58)	33.33 (13/39)
Birth weight (g), mean ± SD	3049.4 ± 650.2	3225.7 ± 445.6	2887.9 ± 805.4	3119.4 ± 686.3	3043.8 ± 688.9	2818.7 ± 822.2*
Low birth weight rate, < 2500 g , % (*n*)	17.89% (102/570)	8.70% (2/23)	30.77% (4/13)	11.43% (8/70)	17.33% (13/75)	40.38%(21/52)***
Very low birth weight rate, < 1500 g, % (*n*)	1.75% (10/570)	0.00% (0/23)	7.70% (1/13)	1.43% (1/70)	1.33% (1/75)	5.77% (3/52)
Gestational age at diagnosis (w), mean ± SD	37.7 ± 2.3	38.4 ± 1.6	37.0 ± 4.1	37.6 ± 2.5	38.1 ± 1.5	37.4 ± 3.2
Preterm birth rate, <37 weeks,% (*n*)	20.65 (95/460)	4.55 (1/22)*	10.00 (1/10)	19.30 (11/57)	15.52 (9/58)	20.51 (8/39)
Very preterm birth rate, <34 weeks, % (*n*)	5.00 (23/460)	4.55 (1/22)	10.00 (1/10)	5.26 (3/57)	1.72 (1/58)	10.26 (4/39)
Birth height (cm)	48.9 ± 3.1	49.9 ± 1.1	48.8 ± 4.5	49.3 ± 3.5	48.8 ± 3.1	47.2 ± 4.9***
Congenital anomaly birth, *n*	4	0	0	0	0	0

*Note*: Non‐AZF deletion group serves as the reference. Data are presented as median (IQR) for non‐normally distributed variables, or as % (*n*/*N*). Asterisks (*) indicate significant differences compared with the Non‐AZF deletion group. Significance levels: *p ≤ 0.05, **p ≤ 0.01, ***p ≤ 0.001.

**TABLE 5:** Multivariate logistic regression analyses of embryologic outcomes in male patients with or without AZFc deletion or duplication after undergoing ICSI treatment.

Indicators	b2/b4 deletion	b1/b3 deletion	b2/b3 deletion	gr/gr deletion	Duplication only
Fertilization rate per oocytes retrieval					
Unadjusted OR (95% CI)	0.65 (0.45, 0.95)*	1.09 (0.63, 1.87)	1.03 (0.77, 1.37)	1.01 (0.75, 1.37)	0.87 (0.59, 1.28)
Adjusted OR (95% CI)	0.63 (0.45, 0.88)**	0.90 (0.56, 1.46)	0.99 (0.74, 1.32)	0.96 (0.70, 1.32)	0.88 (0.60, 1.30)
2PN fertilization rate					
Unadjusted OR (95% CI)	0.67 (0.47, 0.93)*	1.32 (0.78, 2.23)	1.07 (0.82, 1.40)	1.12 (0.86, 1.47)	0.98 (0.68, 1.41)
Adjusted OR (95% CI)	0.65 (0.47, 0.89)**	1.10 (0.70, 1.74)	1.03 (0.79, 1.34)	1.07 (0.82, 1.41)	0.96 (0.66, 1.38)
2PN cleavage rate					
Unadjusted OR (95% CI)	1.88 (0.49, 7.16)	2.60 (0.38, 17.83)	3.14 (0.94, 10.45)	0.58 (0.31, 1.09)	0.82 (0.37, 1.81)
Adjusted OR (95% CI)	1.75 (0.50, 6.18)	1.77 (0.22, 14.30)	3.51 (0.98, 12.52)	0.66 (0.30, 1.45)	0.82 (0.36, 1.87)
High‐quality embryo rate					
Unadjusted OR (95% CI)	0.79 (0.53, 1.17)	1.09 (0.79, 1.52)	0.97 (0.77, 1.23)	1.03 (0.79, 1.34)	0.97 (0.72, 1.31)
Adjusted OR (95% CI)	0.80 (0.54, 1.19)	1.01 (0.68, 1.49)	1.00 (0.79, 1.27)	1.08 (0.82, 1.42)	0.98 (0.72, 1.33)
Blastocyst formation rate					
Unadjusted OR (95% CI)	0.62 (0.40, 0.95)*	0.70 (0.44, 1.11)	1.24 (0.97, 1.60)	0.98 (0.74, 1.29)	0.91 (0.66, 1.25)
Adjusted OR (95% CI)	0.67 (0.43, 1.05)	0.59 (0.32, 1.06)	1.20 (0.93, 1.56)	1.03 (0.78, 1.37)	0.90 (0.63, 1.27)

*Note*: To assess differences and similarities between the non‐AZF deletion and the AZFc deletion or duplication groups respectively, the non‐AZF deletion group was taken as the reference. OR = odds ratio, after adjusting for the following confounding factors: female BMI, duration of infertility, female AMH, metaphase II oocytes, male age, male BMI, seminal concentration, the semen type, the sperm extraction method, and basal antral follicle count. Asterisks (*) indicate significant differences: *p ≤ 0.05, **p ≤ 0.01, ***p ≤ 0.001.

Abbreviation: CI, confidence interval.

**TABLE 6:** Multivariate logistic regression analyses of cumulative reproductive outcomes from one complete cycle in male patients with or without AZFc deletion or duplication after undergoing ICSI treatment.

Indicators	b2/b4 deletion	b1/b3 deletion	b2/b3 deletion	gr/gr deletion	Duplication only
Implantation rate					
Unadjusted OR (95% CI)	0.71 (0.40, 1.25)	2.30 (1.10, 4.82)*	1.30 (0.90, 1.88)	1.34 (0.94, 1.93)	0.73 (0.49, 1.08)
Adjusted OR (95% CI)	0.62 (0.44, 0.88)**	2.02 (0.91, 4.53)	1.05 (0.73, 1.50)	1.29 (0.92, 1.81)	0.77 (0.58, 1.03)
Biochemical pregnancy rate per transfer					
Unadjusted OR (95% CI)	1.01 (0.48, 2.10)	1.02 (0.32, 3.25)	1.25 (0.74, 2.11)	1.15 (0.67, 1.97)	0.71 (0.43, 1.18)
Adjusted OR (95% CI)	0.78 (0.39, 1.58)	0.89 (0.33, 2.38)	1.01 (0.58, 1.75)	0.85 (0.51, 1.43)	0.77 (0.48, 1.23)
Clinical pregnancy rate per transfer					
Unadjusted OR (95% CI)	0.78 (0.42, 1.43)	1.11 (0.34, 3.58)	1.07 (0.68, 1.68)	1.33 (0.81, 2.17)	0.67 (0.42, 1.07)
Adjusted OR (95% CI)	0.61 (0.36, 1.04)	0.98 (0.35, 2.77)	0.79 (0.51, 1.25)	1.03 (0.64, 1.65)	0.64 (0.41, 0.99)*
Ongoing pregnancy rate per transfer					
Unadjusted OR (95% CI)	0.98 (0.54, 1.77)	0.72 (0.32, 1.63)	1.09 (0.70, 1.70)	1.32 (0.85, 2.04)	0.69 (0.44, 1.08)
Adjusted OR (95% CI)	0.88 (0.50, 1.54)	0.68 (0.32, 1.43)	0.80 (0.53, 1.19)	1.12 (0.75, 1.68)	0.74 (0.48, 1.14)
Miscarriage rate per pregnancy					
Unadjusted OR (95% CI)	0.86 (0.39, 1.88)	2.17 (1.00, 4.69)*	1.01 (0.65, 1.56)	0.90 (0.57, 1.44)	1.26 (0.80, 2.00)
Adjusted OR (95% CI)	1.09 (0.49, 2.40)	2.64 (0.95, 7.32)	1.32 (0.86, 2.03)	0.95 (0.57, 1.58)	1.14 (0.68, 1.93)
Live birth rate per transfer					
Unadjusted OR (95% CI)	0.92 (0.48, 1.77)	0.63 (0.27, 1.48)	1.14 (0.73, 1.78)	1.32 (0.84, 2.06)	0.72 (0.46, 1.13)
Adjusted OR (95% CI)	0.82 (0.45, 1.47)	0.51 (0.23, 1.15)	0.84 (0.56, 1.27)	1.11 (0.72, 1.72)	0.80 (0.51, 1.25)
Biochemical pregnancy rate per woman					
Unadjusted OR (95% CI)	0.88 (0.26, 3.01)	1.27 (0.16, 9.95)	0.74 (0.35, 1.57)	0.65 (0.31, 1.33)	0.71 (0.31, 1.65)
Adjusted OR (95% CI)	0.65 (0.17, 2.47)	0.89 (0.09, 8.55)	0.54 (0.23, 1.28)	0.44 (0.19, 1.04)	0.91 (0.34, 2.42)
Clinical pregnancy rate per woman					
Unadjusted OR (95% CI)	0.91 (0.31, 2.71)	0.84 (0.18, 3.85)	0.76 (0.39, 1.48)	0.93 (0.46, 1.89)	0.60 (0.29, 1.20)
Adjusted OR (95% CI)	0.66 (0.21, 2.11)	0.52 (0.09, 3.08)	0.51 (0.24, 1.08)	0.70 (0.31, 1.58)	0.61 (0.27, 1.35)
Ongoing pregnancy rate per woman					
Unadjusted OR (95% CI)	1.13 (0.42, 3.02)	1.35 (0.29, 6.15)	0.93 (0.51, 1.70)	1.20 (0.63, 2.31)	0.64 (0.34, 1.19)
Adjusted OR (95% CI)	0.78 (0.27, 2.24)	1.14 (0.19, 6.87)	0.62 (0.31, 1.25)	1.18 (0.54, 2.56)	0.78 (0.37, 1.62)
Live birth rate per woman					
Unadjusted OR (95% CI)	0.92 (0.36, 2.31)	0.83 (0.23, 3.08)	0.95 (0.52, 1.74)	1.12 (0.59, 2.11)	0.65 (0.35, 1.22)
Adjusted OR (95% CI)	0.62 (0.23, 1.68)	0.53 (0.10, 2.64)	0.63 (0.32, 1.27)	1.04 (0.49, 2.22)	0.82 (0.39, 1.71)

*Note*: To assess differences and similarities between the non‐AZF deletion and the AZFc deletion or duplication groups, the non‐AZF deletion group was taken as the reference. OR = odds ratio, after adjusting for the following confounding factors: female age, female BMI, duration of infertility, female AMH, basal antral follicle count, metaphase II oocytes, male age, male BMI, seminal concentration, the semen type, the sperm extraction method, number of transferred cycles and number of embryos transferred. Asterisks (*) indicate significant differences: *p ≤ 0.05, **p ≤ 0.01, ***p ≤ 0.001.

Abbreviation: CI, confidence interval.

Compared with the control group, the b2/b4 deletion group exhibited a suboptimal embryologic outcome after ICSI treatment, which was characterized by a significantly lower fertilization rate per oocyte retrieval (72.22% vs. 79.89%; adjusted OR, 0.63; 95% CI, 0.45–0.88, *p* < 0.01) and 2PN fertilization rate (66.37% vs. 74.80%; adjusted OR, 0.65; 95% CI, 0.47–0.89, *p* < 0.01) before and after adjustment for potential confounding factors. These adjustment factors included female age, female BMI, duration of infertility, female AMH, MII oocytes, male age, male BMI, seminal concentration, semen type, sperm extraction method, and bAFC. The implantation rate in the b2/b4 deletion group was significantly lower than that in the control group (44.26% vs. 51.91%; adjusted OR, 0.62; 95% CI, 0.44–0.88, *p* < 0.01). After adjustment for the above factors, the number of transferred cycles, and the number of embryos transferred, the multivariate logistic regression analyses showed that there were no statistically significant differences in other embryologic and cumulative reproductive outcomes between the b2/b4 deletion group and the control group. However, the key indicators in the b2/b4 deletion group were notably lower than those in the control group. These indicators include the high‐quality embryo rate (46.70% vs. 52.53%, unadjusted OR, 0.79; adjusted OR, 0.80), blastocyst formation rate (50.00% vs. 62.24%, unadjusted OR, 0.62; adjusted OR, 0.67), the live birth rate per transferred embryo (55.00% vs. 57.00%, unadjusted OR, 0.92; adjusted OR, 0.82), and live birth rate per woman(78.57% vs. 80.00%, unadjusted OR, 0.92; adjusted OR, 0.62), with the exception of the 2PN cleavage rate. Overall, b2/b4 deletion might have an adverse effect on the fertilization, implantation, clinical pregnancy, and live birth rates per initiated treatment cycle.

There were no significant differences in fertilization rate per oocyte retrieval and 2PN fertilization rate between three partial AZFc deletion groups (b1/b3, b2/b3, and gr/gr deletions) and the control group before and after adjustment for confounding factors. This suggests that these partial deletions did not have a significant effect on the fertilization rate after ICSI treatment. However, other indicators related to embryologic and cumulative reproductive outcomes varied among the groups. Specifically, the miscarriage rate per pregnancy was higher in the b1/b3 deletion group compared with the control group (33.33% vs. 13.04%, unadjusted OR, 2.17; 95% CI, 1.0–4.69, *p* < 0.05), indicating an obviously statistically significant difference before adjustment for confounding factors. In contrast, the 2PN cleavage rate was higher in the b2/b3 deletion group and lower in the gr/gr deletion group compared with the control group (99.47% vs. 98.40%, *p* < 0.05; 97.36% vs. 98.40%, *p* < 0.001). Additionally, the blastocyst formation rate was higher in the b2/b3 deletion group compared with the control group (67.16% vs. 62.24%, *p* < 0.05). The implantation rate (59.21% vs. 51.91%, *p* < 0.05) and the number of cycles without a transfer (2.82% vs. 0.52%, *p* < 0.05) were significantly higher in the gr/gr deletion group compared with the control group. The multivariate regression analysis revealed that the live birth rate per woman was lower in the b1/b2 deletion group and b2/b3 deletion group compared with the control group. However, there was no discernible difference in the live birth rate per woman between the gr/gr deletion group and the control group.

In the partial AZFc duplication group, six types of typical primary gene duplications predicted by NAHR were identified, as detailed in Table 1. Among these types of gene duplications, the gr/gr duplication was most prevalent, accounting for 55.56%. The seminal phenotype of individuals with primary AZFc duplication varied from azoospermia to normozoospermia. The embryologic and cumulative reproductive outcomes in the primary AZFc duplication group were inferior to those in the control group. Furthermore, the multivariate logistic regression analyses after adjusting for relevant indicators revealed that the primary AZFc duplication group exhibited a notably lower clinical pregnancy rate per transferred embryo (56.25% vs. 65.97%; adjusted OR, 0.64; 95% CI, 0.41–0.99, *p* < 0.05) compared with the control group. In addition, the high‐quality embryo rate, clinical pregnancy rate, and live birth rate in the primary AZFc duplication group were inferior to those in the control group.

Table 4 also presents the obstetric and neonatal outcomes, revealing no significant differences among the groups in terms of time to first positive hCG, time to first clinical pregnancy, time to first ongoing pregnancy, and time to first live birth. Notably, the b2/b4 deletion group showed a significantly higher rate of singleton pregnancies (95.45% vs. 76.09%, *p* < 0.05) and a lower rate of preterm deliveries (4.55% vs. 20.65%, *p* < 0.05) compared with the control group. Furthermore, the neonatal outcomes, specifically birth weight (mean ± SD, 2818.7 ± 822.2 g vs. 3049.4 ± 650.2 g, *p* < 0.05) and birth height (mean ± SD, 47.2 ± 4.9 cm vs. 48.9 ± 3.1 cm, *p* < 0.001), were significantly lower in the partial AZFc duplication group compared with the control group. Additionally, the partial AZFc duplication group showed a significantly higher rate of low birth weight (40.4% vs. 17.9%, *p* < 0.001) compared with the control group. Only four cases of congenital anomaly birth were detected in the control group.

In addition to the above findings, we conducted further analysis to compare the ICSI embryology and the neonatal outcomes between the subtypes of primary AZFc duplication groups and the non‐AZF deletion group (Tables S3–S7). The patients with b2/b3 duplication showed a significantly higher sperm concentration compared with the control group (26.30 vs. 5.75 × 10^6^/mL, *p* < 0.05). However, the fertilization rate was significantly lower in patients with b2/b3 duplication (74.17% vs. 79.89%, *p* < 0.05) compared with the control group. The most notable finding was observed in neonatal outcomes, and the patients with gr/gr and b2/b3 duplications showed significantly higher rates of low birth weight (<2500 g) compared with the control group (42.42% and 41.18% vs. 17.89%, *p* < 0.001 and *p* < 0.01, respectively). The multivariate logistic regression analysis revealed that the association between AZFc duplication (both gr/gr and b2/b3 duplications) and low birth weight rate remained significant after adjusting for potential confounding factors, with elevated adjusted ORs (gr/gr: adjusted OR, 7.91, 95% CI, 2.66–23.55, *p* < 0.001; b2/b3: adjusted OR, 5.59, 95% CI, 1.46–21.50, *p* < 0.05). This trend was particularly evident in twin pregnancies, especially for the patients with the gr/gr duplication group (75.00% vs. 37.73%, *p* < 0.01).

## 4 DISCUSSION

In the present study, we carried out a systematic retrospective analysis of the effects of AZFc deletion or duplication on the assisted reproductive outcomes in patients in northeastern China who underwent their first ICSI cycles. The precise screening of Y chromosome microdeletions based on next‐generation sequencing technology can benefit the patients in identifying their male infertility etiology. Recently, a number of studies have investigated the effects of b2/b4 deletion on the embryologic and pregnant outcomes after ICSI treatment,^14^, ^15^, ^17^, ^18^, ^25^ but the effects of AZFc deletions and duplication on assisted reproduction outcomes remain unclear. This study demonstrated that there was a statistically significant decrease in the fertilization rate and 2PN fertilization rate in the b2/b4 deletion group compared with the control group. In addition, key indicators such as high‐quality embryo rate, blastocyst formation rate, live birth rate per transferred embryo, and live birth rate per woman were critically lower than those in the control group. These results align with those of previous studies by Zhang et al.^14^ and Andrew et al,^26^ which reported adverse effects of AZFc deletions on ICSI outcomes, including lower rates of fertilization, clinical pregnancy, and live birth. The consistent results across studies strengthen the evidence base for the negative effects of Y chromosome microdeletion on assisted reproductive outcomes.

Given the poor outcomes in men with complete AZFc deletion after ICSI treatment, we investigated the effects of three partial AZFc deletions (b1/b3 deletion, b2/b3 deletion, gr/gr deletion) on the embryologic and pregnant outcomes after ICSI treatment. Our findings showed no significant differences in fertilization rate between the three partial AZFc deletion groups and the control group, in contrast with results from the complete AZFc deletion group. This inconsistency may be attributed to poor sperm function in patients with b2/b4 deletion, which can affect embryonic development at early stages, such as the fertilization stage, resulting in the inability to activate oocytes and reduced fertilization rates.^27^ Specifically, the average sperm concentration in the b2/b4 deletion group was significantly lower than those in the other groups, and the proportion of patients with azoospermia and severe oligozoospermia (equal to 97%) was significantly higher than those in other groups. A study has indicated that the incidence rates of DNA fragmentation, mitochondrial dysfunction, and chromosomal aneuploid are significantly higher in the spermatozoa of oligoasthenozoospermic males,^28^ In addition, DNA damage in the male germ line is identified as a risk factor for various adverse clinical outcomes such as poor semen quality, low fertilization rate, impaired pre‐implantation embryo development, and miscarriage, with an increased risk of morbidity in the offspring.^29^ These patients with b2/b4 deletion in poor‐quality spermatozoa are considered to be possible reasons for fertilization failure.^30^ Similarly, it was found by Wei et al^31^ that oligoasthenozoospermic men have a lower fertilization rate, poor embryo quality, and a lower pregnancy rate in comparison with normozoospermic men. Zhou et al.^12^ conducted the first systematic review and meta‐analysis comparing ART outcomes between testicular and ejaculated spermatozoa in infertile men with AZFc deletions. Their analysis found no significant differences in major reproductive outcomes between the two groups. In our study, because of the sample size limitation, the sperm extraction method was just used to adjust for confounding factors, and no further stratified analysis was performed.

The AZFc region is rich in amplicons. Therefore, it is predisposed to a series of rearrangements such as deletion or duplication, and deletion combined with duplication.^32^ The effects of these rearrangements in spermatogenesis are controversial and variable among different human populations.^33^ Recent population‐based association studies suggest that several recurring partial AZFc deletions (b1/b3, b2/b3, and gr/gr deletions with or without secondary duplication) and primary AZFc duplications (duplication only, without deletion) may impair spermatogenesis.^5^, ^6^, ^34^, ^35^, ^36^, ^37^, ^38^, ^39^, ^40^ Recent reports indicate that secondary duplications following partial AZFc deletions have the potential to restore the total motile sperm count to a normal level.^41^ While a multicentre study of the European population showed that the sperm production in males with gr/gr deletion combined with duplication is lower than that in those with gr/gr deletion only.^40^ Yang et al.^9^ proposed that multiple duplications within the partially deleted AZFc region may have a more pronounced negative effect on spermatogenesis, resulting in a greater decrease in sperm production compared with other types of partial AZFc deletion such as gr/gr deletion and b2/b3 deletion. The study conducted by Lu et al.^35^ in Han Chinese populations demonstrated that additional AZFc duplications do not serve as a compensatory mechanism for generating spermatozoa, but indicate an increased susceptibility to spermatogenic impairment in individuals with b2/b3 deletion. In this study, the embryologic and cumulative reproductive outcomes varied among three partial AZFc deletion groups, Notably, there was a higher miscarriage rate per pregnancy in the b1/b3 deletion group than in the control group. The primary endpoint was defined as the live birth rate per woman. Among three partial AZFc deletion groups, the live birth rate per woman after the first ICSI treatment cycle was lower in the b1/b2 and b2/b3 deletion groups, while no significant effect on the live birth rate per woman after the first ICSI treatment cycle was observed in the gr/gr deletion group compared with the control group. This disparity may be attributed to variations in the frequency of complex CNVs (deletion + duplication) among the three deletions (69.23% in the b1/b3 deletion group, 41.67% in the b2/b3 deletion group, and 14.08% in the gr/gr deletion group). The combined presence of genomic deletion and duplication appears to have a more pronounced effect on sperm count compared with the isolated genomic deletion. This suggests that complex CNVs, such as deletion + duplication, may have an exacerbated effect on embryonic development and clinical pregnancies. This can explain why some partial deletion groups, such as the b1/b3 deletion group, experience a worse reproductive outcome than others, despite having a similar fertilization rate. Further researches are needed to generate high‐quality evidence‐based medical knowledge in this area.

The wide range of seminal phenotypes observed in patients with AZFc duplication, from azoospermia to normozoospermia, suggests that these AZFc duplications can impair spermatogenesis to varying degrees. On a theoretical basis, the genomic duplication in the AZF region can affect male fertility, either leading to an excess of gene products or causing an imbalance among genes involved in spermatogenesis.^42^ Lu et al.^35^ have suggested that increased expression of certain genes in the duplicated AZFc region may interfere with normal spermatogenesis. Yang et al.^9^ cautiously suggested that the association between AZFc duplication or deletion and spermatogenesis failure cannot be ascribed solely to changes in gene dosage, the effects of destructive chromatin configuration, Y chromosome linage, and gene interaction/imbalance in AZFc should also be considered in investigating the effect of the AZFc structure.^1^ One latest study reported two cases of patients with genomic duplications spanning 4.8 Mb and 6.2 Mb in AZFc region, both patients with normozoospermia had offsprings with their spouses successfully.^43^ Therefore, the effects of primary AZFc duplication on spermatogenesis remain controversial, and the underlying reasons for these discrepant outcomes are still unknown.

One of the most significant findings in this study is the detrimental effect of primary AZFc duplications on ICSI outcome, especially the outcome of neonates during the perinatal period. Our findings suggest that AZFc duplications (including gr/gr and b2/b3 duplication) can significantly increase the risk of low birth weight in neonates delivered through ICSI treatment, particularly in twin neonates. This risk became more pronounced after adjustment for confounding factors, highlighting a potential causal relationship between primary AZFc duplications and low birth weight. Additionally, AZFc duplications are associated with a significant reduction in neonatal birth length, with this effect also being more pronounced in twin births. Interestingly, despite these differences, most reproductive outcomes such as clinical pregnancy rate and live birth rate after adjustment for confounding factors did not differ significantly between the primary AZFc duplication group and the control group. This might suggest that the effect of AZFc duplication on early embryo development is relatively limited, but AZFc duplication may affect fetal growth through effects on placental function or other mechanisms. These findings underscore the importance of paying attention to the effects of AZFc duplications on neonatal perinatal safety, particularly the risk of low birth weight in neonates resulting from ICSI‐assisted reproduction. Future studies should explore the potential mechanisms by which AZFc duplications affect fetal growth and the possible long‐term health consequences for children.

Our study on the effects of AZFc deletions on ART outcomes aligns with the meta‐analysis of Colaco et al.^44^ in demonstrating adverse impacts, although it differs in methodology and findings. Colaco et al.^44^ investigated the effect of complete AZFc deletion (b2/b4) on ART outcomes by considering IVF and ICSI as a combined group, whereas our study focused exclusively on the effects of CNVs in the AZFc region on ICSI outcomes. Both studies consistently showed that b2/b4 deletion can significantly reduce fertilization rate, 2PN fertilization rate, clinical pregnancy rate, and live birth rates compared with the control group, underscoring the crucial role of Y chromosome integrity in achieving successful ART outcomes. However, regarding cleavage rates, our study did not observe a statistically significant difference, even though the deletion group exhibited a higher 2PN cleavage rate compared with the control group. In contrast, Colaco et al.^44^ reported that the cleavage rate was significantly lower in the AZFc complete deletion group than in the control group. This discrepancy may be because of differences in calculation methods and study populations. Furthermore, this study conducted an in‐depth analysis of the AZFc subregions and investigated the effects of CNVs in the AZFc region on offspring outcomes after ICSI treatment, which is an aspect not addressed in the study by Colaco et al.^44^ Additionally, Colaco et al.^44^ compared the sperm retrieval rate between the deletion group and the nondeletion group using microdissection testicular sperm extraction, which is a variable not explored in this study. In this study, the source of spermatozoa was treated solely as a confounding variable.

To address the challenges associated with NGS‐based approaches for AZF CNV analysis, we implemented a rigorous validation process, as detailed in our previously published literature.^20^ During the initial establishment of the capture technique in NGS, we conducted extensive validations in 326 samples, which were collected from the infertile patients, their family members, and sperm bank donors with or without AZF deletion on the Y chromosome detected by conventional polymerase chain reaction of STS (STS‐PCR). Our results showed that NGS had a higher detection rate of CNVs than STS‐PCR; NGSs could identify these CNVs that had been detected by STS‐PCR by 100% and could also identify more CNVs that had not been detected by STS‐PCR. The complex CNVs were detected by qPCR, the results were consistent with those detected by NGS which confirms the the reliability of NGS‐based approach and demonstrates the superior sensitivity of NGS in detecting complex CNVs in the AZF region.

Although NGS approaches for detecting Y chromosome microdeletions have been reported and applied,^42^, ^45^ there are still some methodological limitations, particularly in genomic regions characterized by a high gene copy number and complex rearrangement. To address these limitations, a capture technology was used in this study to design three‐segment probes specifically for the AZF region of the Y chromosome, as described in the methods section. These probes show characteristics such as specificity, complementarity, no self‐complementary sequence, and high GC content. In addition, a stringent and comprehensive bioinformatics pipeline, including rigorous read filtering, precise genome alignment, signal normalization, and advanced statistical analysis, has been applied. This probe design strategy in combination with a rigorous bioinformatics approach can greatly improve the specificity and accuracy of AZF region analysis, thus solving the common problems in sequencing in genomic duplication region. Although challenges remain in dealing with complex rearrangements, the method used in this study provides a more reliable way to detect structural variants in the AZF region of the Y chromosome. Future improvements can be achieved by using methods such as long‐read sequencing or integrating optical mapping to better resolve complex structural variants in these challenging genomic regions.^46^

To our knowledge, this is the first systematic retrospective analysis comparing embryologic and cumulative reproductive outcomes after ICSI treatment between patients with partial AZFc deletion or duplication and controls. Furthermore, this study included a relatively unselected series of couples who were referred to our hospital for undergo their first ART cycles, among them, the women aged below 35 years old with at least five oocytes retrieved, this was designed to avoid the effects of any potential confounding factors such as repeat cycles on ICSI outcomes. The baseline characteristics of all study groups were similar to avoid selection bias. Although there was a significant difference in female BMI between the primary duplication and non‐AZF deletion groups, both were within the normal female BMI range (18.5–24.9 kg/m^2^), which does not reduce the success rate of ART.^47^ Meanwhile, there was a significant difference in the female antral follicle count (AFC) among the b1/b3 deletion, b2/b3 deletion, and non‐AZF deletion groups, but the AFC in all three groups remained within the normal reference range. Additionally, the AMH levels were similar across all groups, with no statistically significant differences from the control group. The differences in AFC among these groups were not sufficient to affect ART outcomes.^48^ The primary outcome was the live birth rate per woman, which was the cumulative live birth delivery rate in the intention to treat the population and a key indicator in measuring the success of IVF/ICSI.^49^ The couples who got the final pregnancy outcome after undergoing fresh and frozen‐thawed embryo transfer were included in this study and 105 couples who did not get final pregnancy outcomes till now were excluded from this study. We performed a thorough follow‐up for all included patients with complete information, and none of the patients were lost to follow‐up. This study has two limitations: first, the study cohort consisted only of the couples undergoing ICSI treatment, which might not accurately represent the broader population of infertile couples who might decide not to pursue treatment or opt for alternative methods; second, this was a single‐center study, and the participants were limited to individuals from major areas in northeast China. Considering the regional limitations, in future research, the sample size will be expanded to conduct a multicenter study to verify the above conclusions.

In conclusion, this study demonstrates that the b2/b4 deletion has a negative effect on the ICSI outcome, especially the fertilization rate and 2PN fertilization rate. Nevertheless, three partial AZFc deletions (b1/b3, b2/b3, and gr/gr deletions with or without secondary duplication) do not have a significant effect on the fertilization rate after ICSI treatment. Additionally, the primary AZFc duplication can lead to the fact that different seminal phenotypes vary from azoospermia to normozoospermia, and may have poor effects on the embryologica and cumulative reproductive outcomes, particularly showing a significant association with low birth weight in neonates after ICSI treatment, these findings are worthy of our attention. In summary, although the utilization of NGS technology for the analysis of Y chromosome microdeletion may not be universally accessible, this study emphasizes the importance of diagnosing Y chromosome microdeletion such as genomic deletion, duplication, and deletion with duplication for preconception genetic counseling. This study offers valuable prognostic insights for infertile couples considering receiving assisted reproductive technologies.

## AUTHOR CONTRIBUTIONS

Ruizhi Liu directed and designed this study. Linlin Li and Xiangyin Liu contributed to the acquisition of clinical data. Xinying Wang and Hongguo Zhang contributed to the analysis of data. Linlin Li and Xiangyin Liu wrote the paper. All authors read and approved the final manuscript.

## CONFLICT OF INTEREST STATEMENT

The authors declare no conflict of interest.

## Supporting information



Supporting Information

## Data Availability

The data that support the findings of this study are available on request from the corresponding author. The data are not publicly available due to privacy or ethical restrictions.
