# Supplementary material for: Effects of AZFc (b2/b4, b1/b3, b2/b3, and gr/gr) deletions and primary duplications on the outcomes of the first intracytoplasmic sperm injection treatment cycle: A single‐center retrospective cohort study
Source: Andrology. 2024 Dec 13;13(7):1757–71. doi: 10.1111/andr.13818 (PMC12476223; doi:10.1111/andr.13818)
Supplement: Supplementary file 1 — Supporting Information [file ANDR-13-1757-s001.docx]

**Supplementary Material**

**Effects of AZFc (b2/b4, b1/b3, b2/b3, and gr/gr) deletions and primary duplications on the outcomes of the first ICSI treatment cycle: A single-center retrospective cohort study**

**Linlin Li, Xiangyin Liu, Xinying Wang, Hongguo Zhang, Ruizhi Liu***

Reproductive Medicine Center, Prenatal Diagnosis Center, First Hospital of Jilin University, Changchun, China

Linlin Li, E-mail: [lll82932404@jlu.edu.cn](mailto:lll82932404@jlu.edu.cn)

Xiangyin Liu, E-mail: xiangyin_liu@jlu.edu.cn

Xinying Wang, E-mail: 673465866@qq.com

Hongguo Zhang, E-mail: zhanghguo@jlu.edu.cn

Ruizhi Liu, E-mail: liurz@jlu.edu.cn

***Correspondence:**

**Ruizhi Liu,** Reproductive Medicine Center, Prenatal Diagnosis Center, First Hospital of Jilin University, No. 1 Xinmin Street, Changchun 130021, China.

Tel and Fax: 86 431 88783909

Email: liurz@jlu.edu.cn

**1 Supplementary figures and tables**

**1.1 Supplementary figures**

**
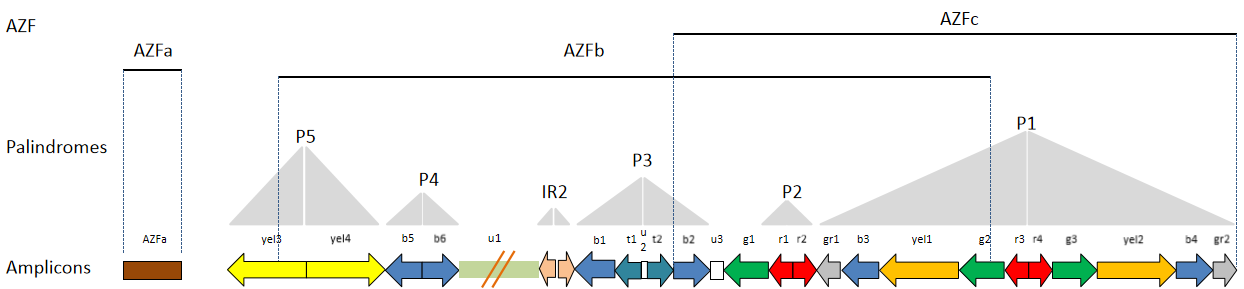
**

Supplementary Figure S1 Schematic diagram of the structure of the AZF region on the Y chromosome

**
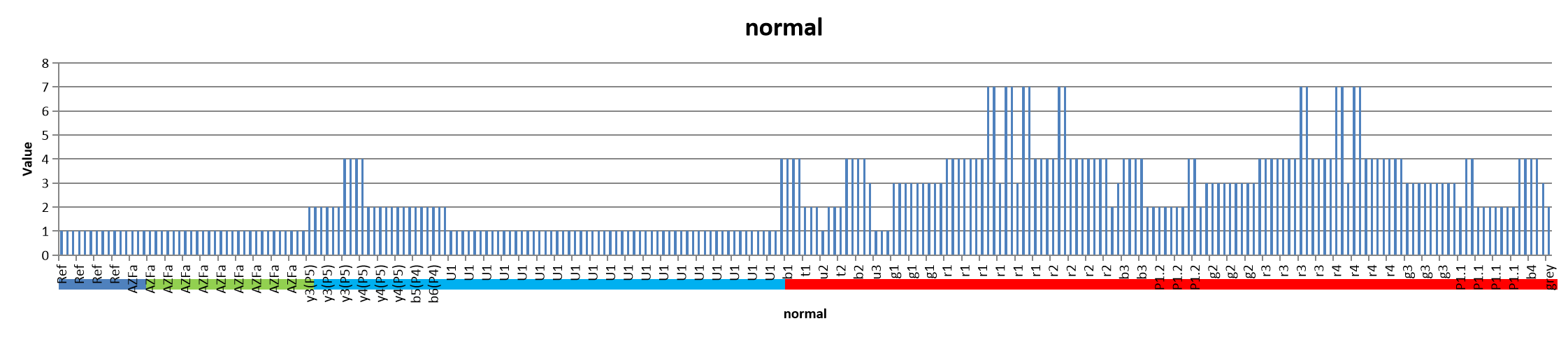
**

Supplementary Figure S2 Probes distribution in the targeted next-generation sequencing (NGS)) utilized in the current study. The employed detection kit comprises a total of 206 specific probes, of which, 165 loci were located within the AZF region, 10 loci located on the short arm of the Y chromosome, 9 loci on the X chromosome, and 22 loci on autosomal chromosomes. Normal refers to male samples with negative NGS detection results.


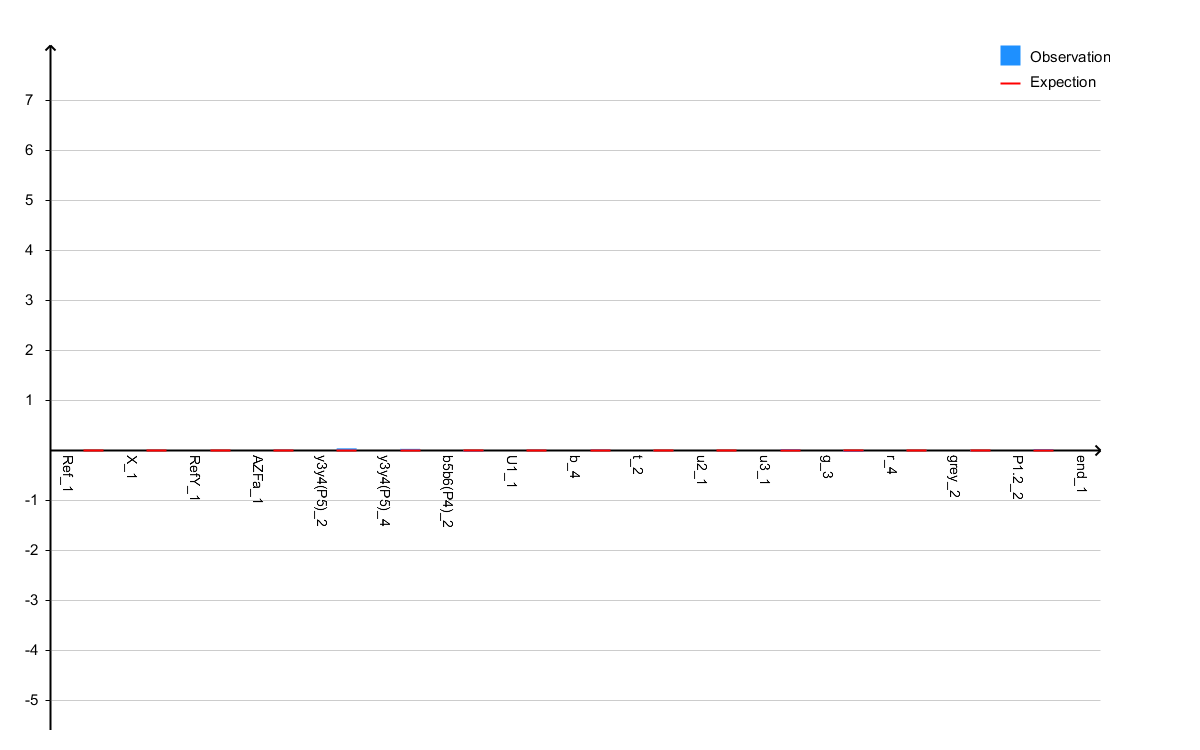


Supplementary Figure S3 The copy number variation(CNV) values in genomic regions from targeted NGS analysis. The X-axis shows genomic regions, and the Y-axis shows CNV values. Blue bars represent observed CNVs, and the red line indicates the expected CNV values at the baseline. Positive values denote the presence of genomic duplications, and negative values denote the presence of genomic deletions. This figure shows that the observed CNV values match the expected CNV values at the baseline in most regions, indicating no changes in copy number and suggesting no deletion in AZF region. Observation refers to observed CNV values. Expection refers to expected CNV values.

**
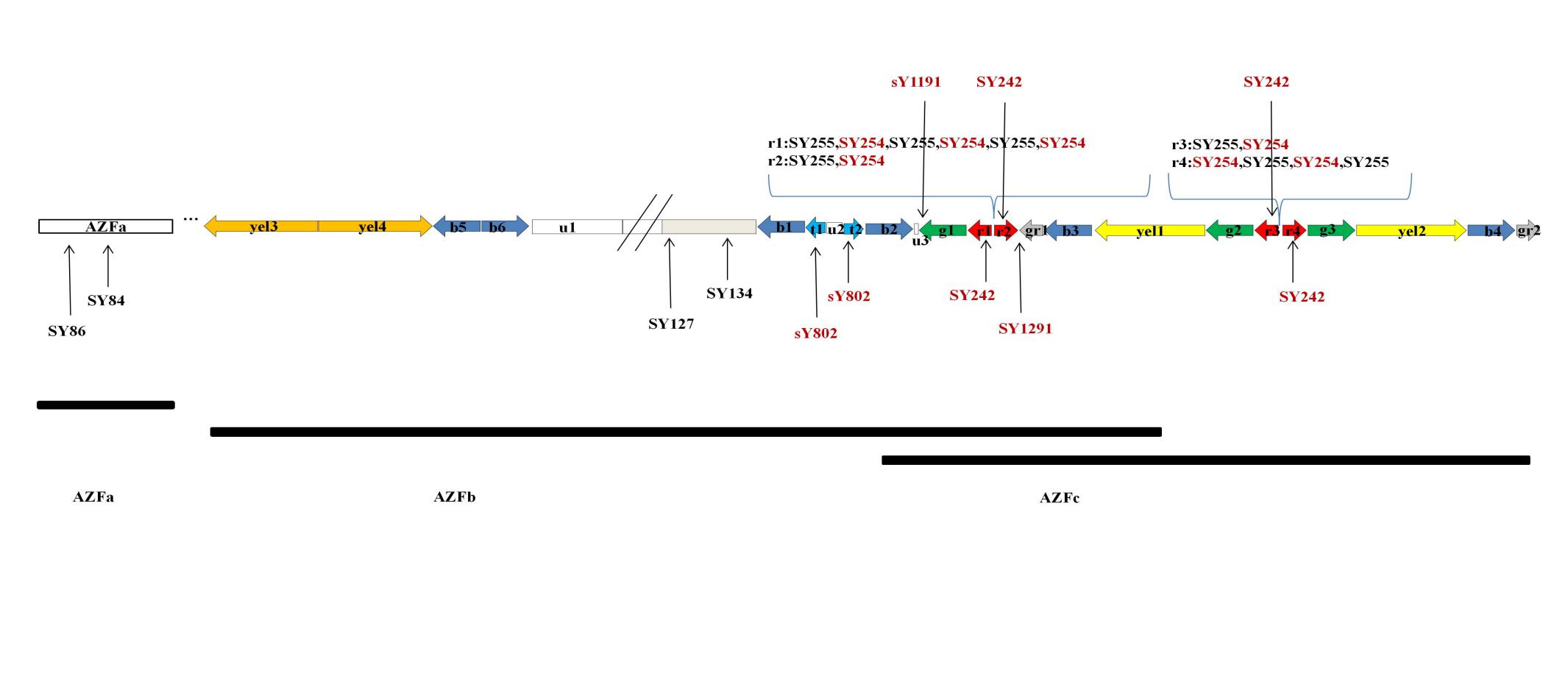
**

Supplementary Figure S4 Schematic diagram of qPCR validation of genomic loci in the AZF region on the Y chromosome.This figure illustrates the localization of the classic 6 STS (sequence-tagged site) markers (SY86, SY84, SY127, SY134, SY254, SY255) and the qPCR validation of genomic loci in the AZF region. The genomic loci validated by qPCR are highlighted in red. Normal copy numbers in healthy individuals are as follows: sY802 (2 copies), sY1191 (1 copy), sY1291 (1 copy), sY254 (7 copies), and sY242 (4 copies). These normal copy numbers serve as the baseline data for detecting genomic deletion and duplication in the AZF region.

**A**

**C**

**B**

**E**

**F**

**D**

**I**

**G**

**H**

**L**

**K**

**J**

**N**

**O**

**M**

**Q**

**P**

Supplementary Figure S5 qPCR validation results of various types of genomic deletion and duplication in the AZFc region. For each CNV to be tested, three samples were examined: two control samples, one male sample(control 1) and one female sample (control 2), both with negative NGS detection results, as well as one randomly selected positive CNV sample. The copy number in the positive CNV sample and control 2 were normalized against the control 1. The qPCR validation results shown in the Supplementary Figure S5 are consistent with the detection sites and expected results listed in Supplementary Table S2: “qPCR validation site selection and expected results for 17 types of AZF deletion and duplication”.

**A: b2/b4 del**


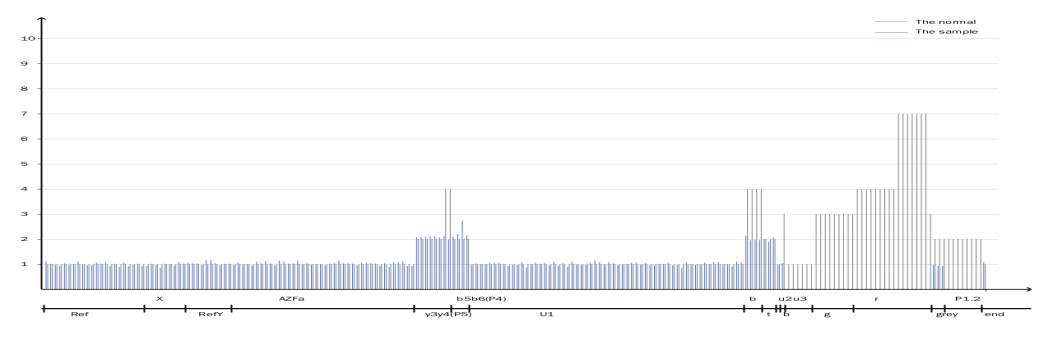
**
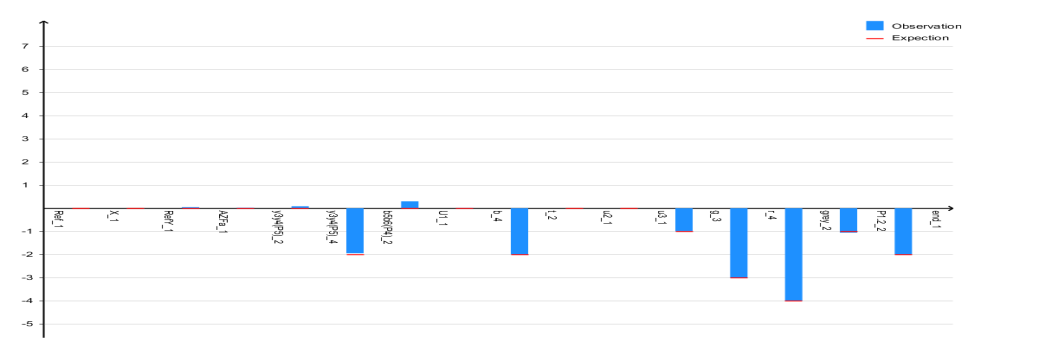
**

**B: b1/b3 del**


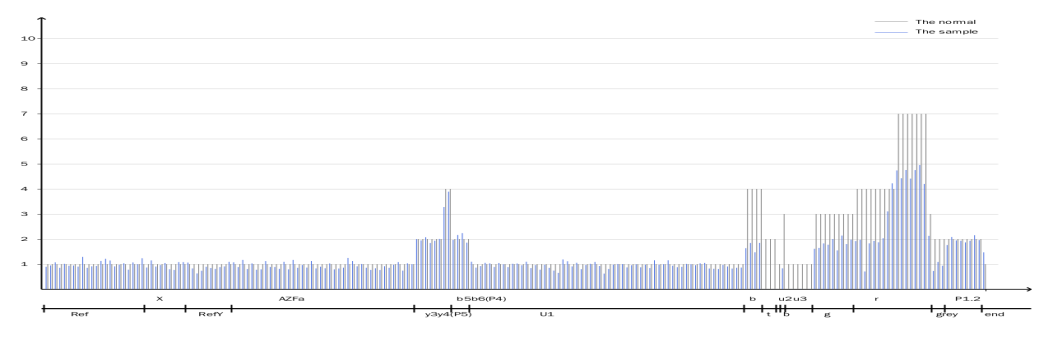

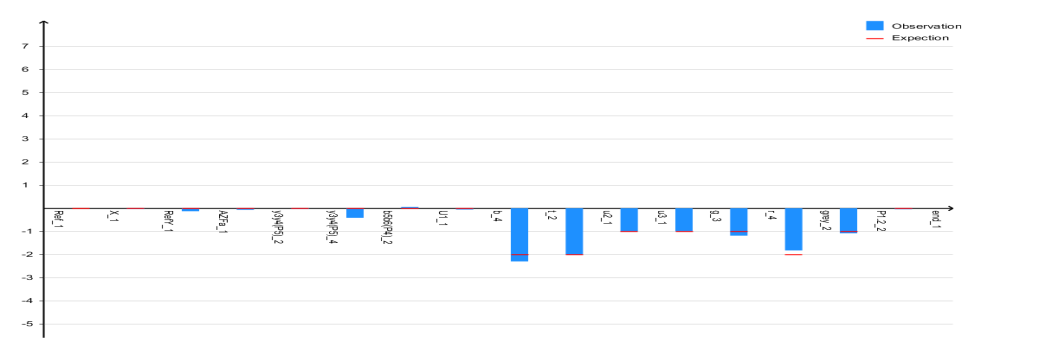


**C: b2/b4 dup+b1/b3del**


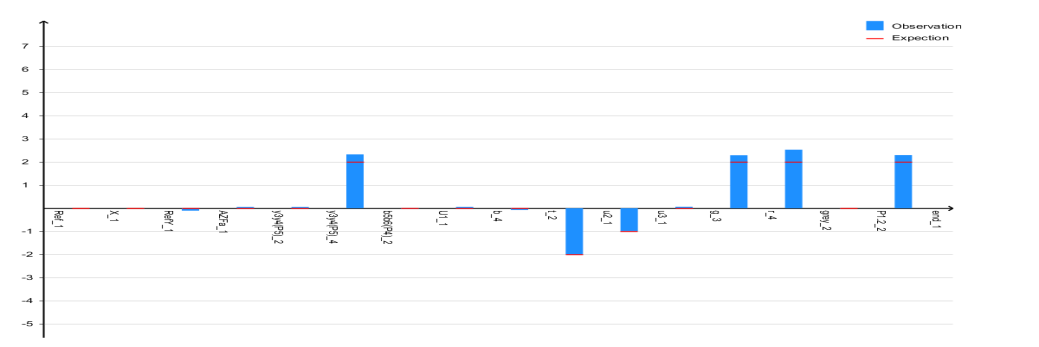

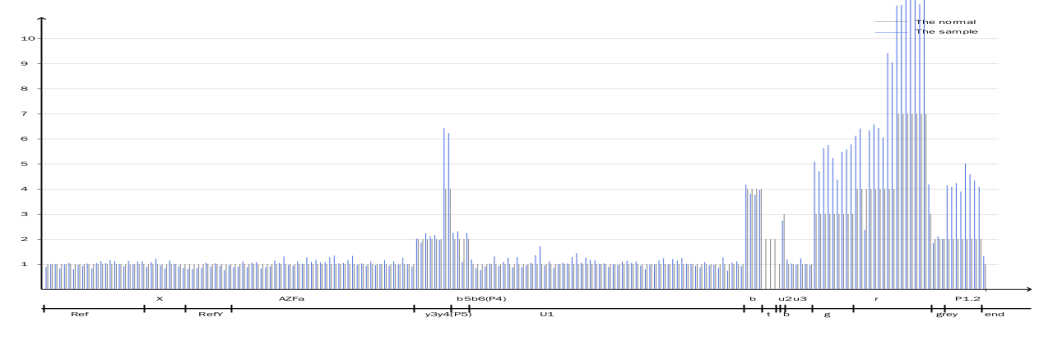


**D: gr/gr dup+b1/b3 del**


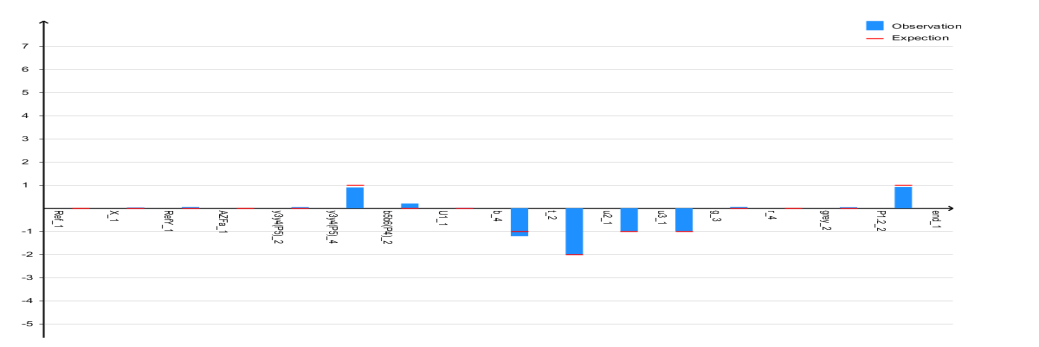

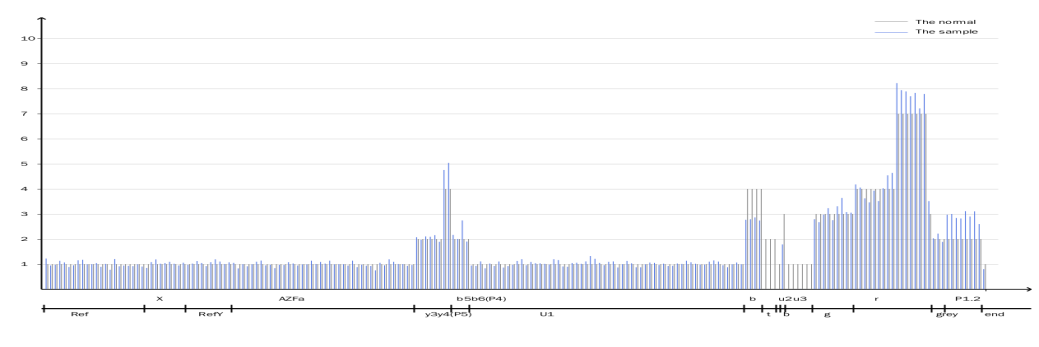


**E: b2/b3 del**


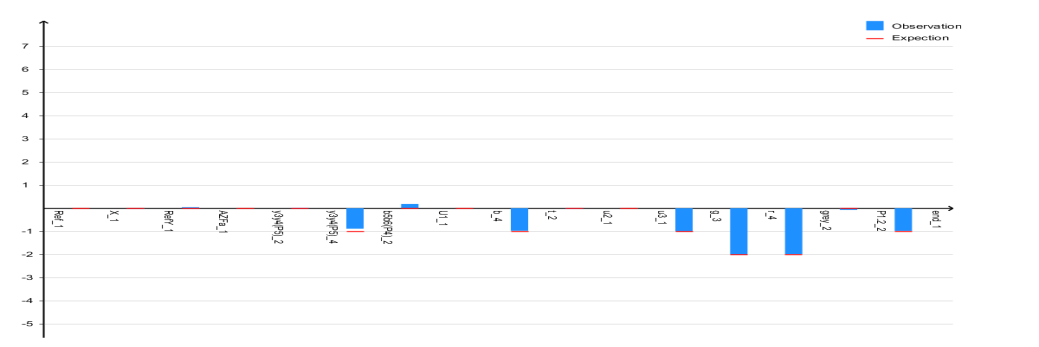

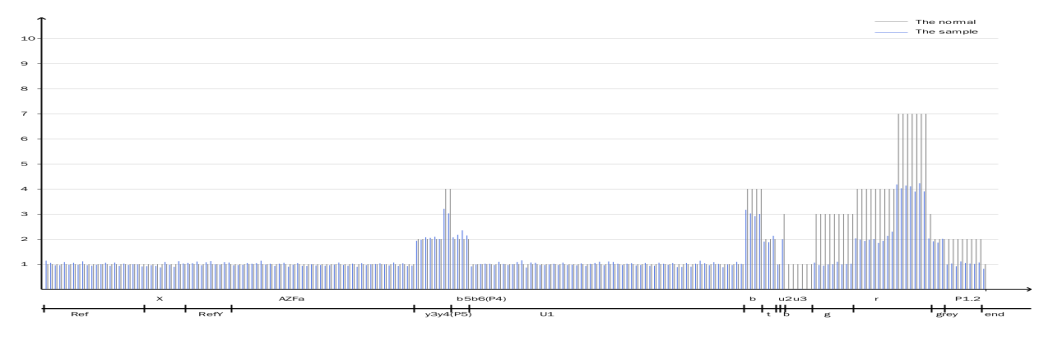


**F: b2/b3 del+b3/b4 dup**


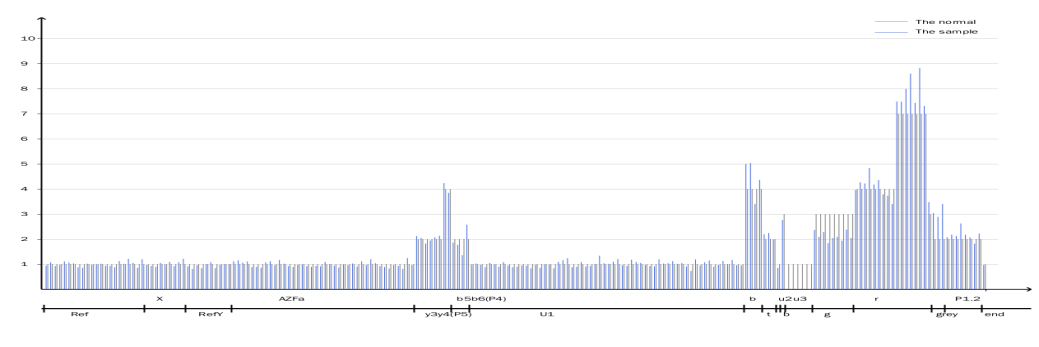

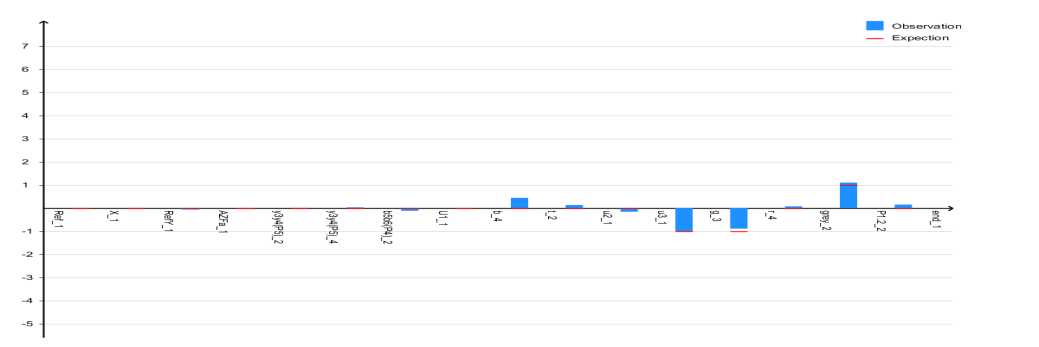


**G: b2/b3 del, grey1 r1 r2 dup**


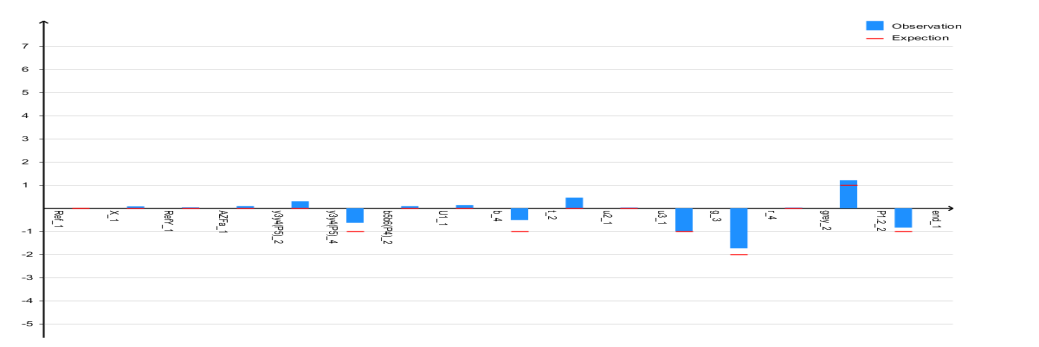

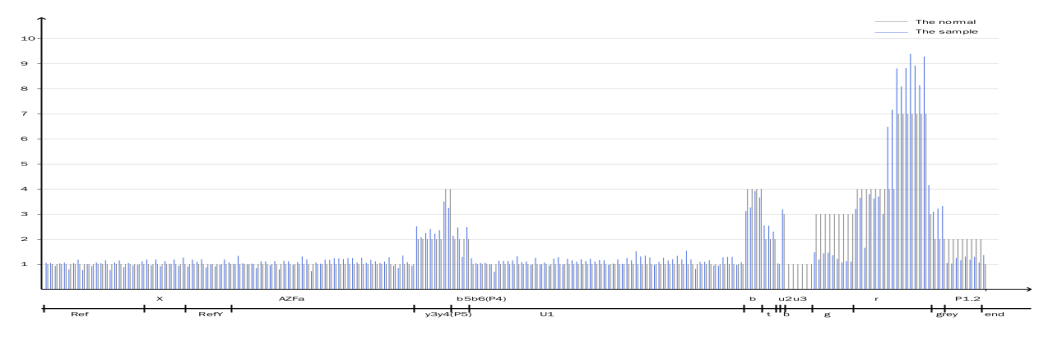


**H: b2/b3 del, grey1 r2 dup**


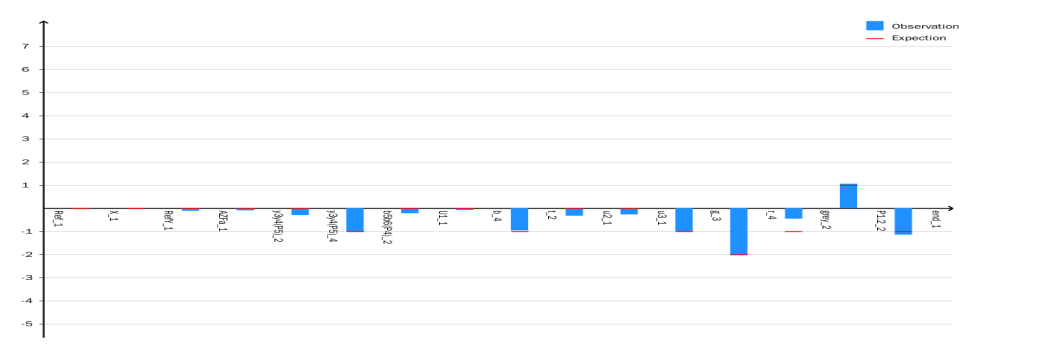

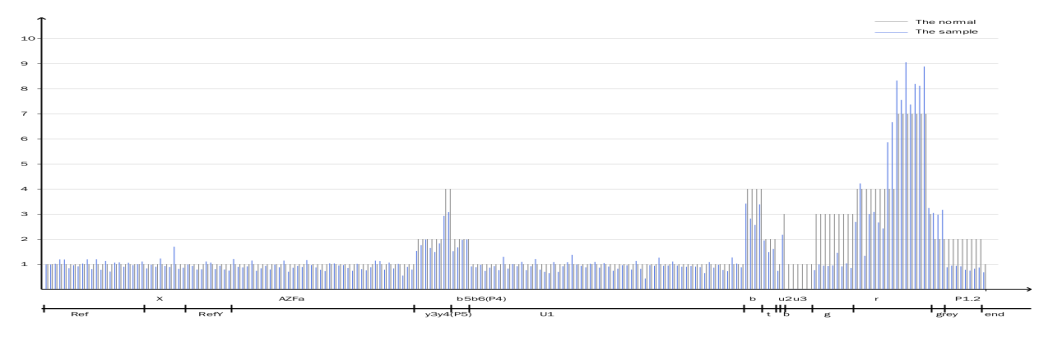


**I: gr/gr del**


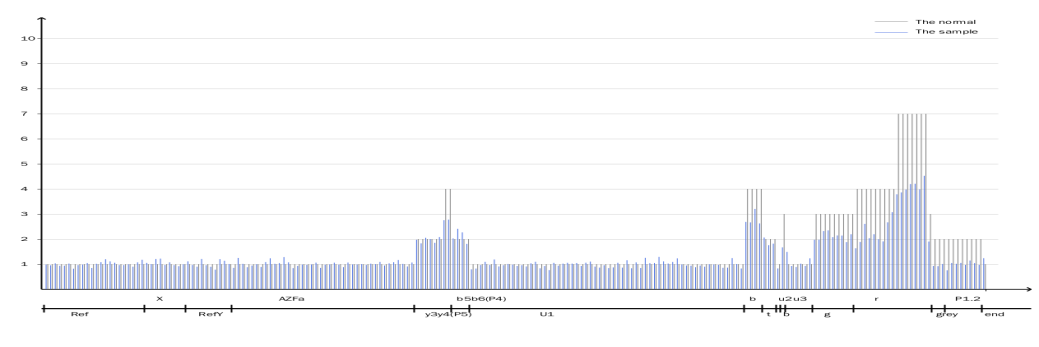

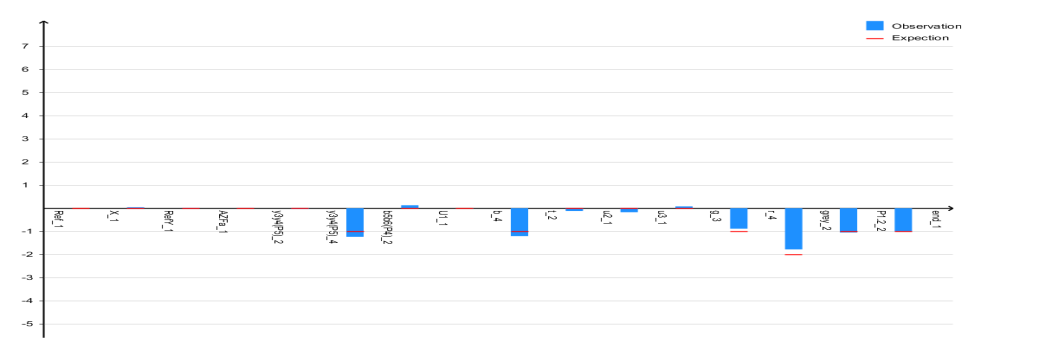


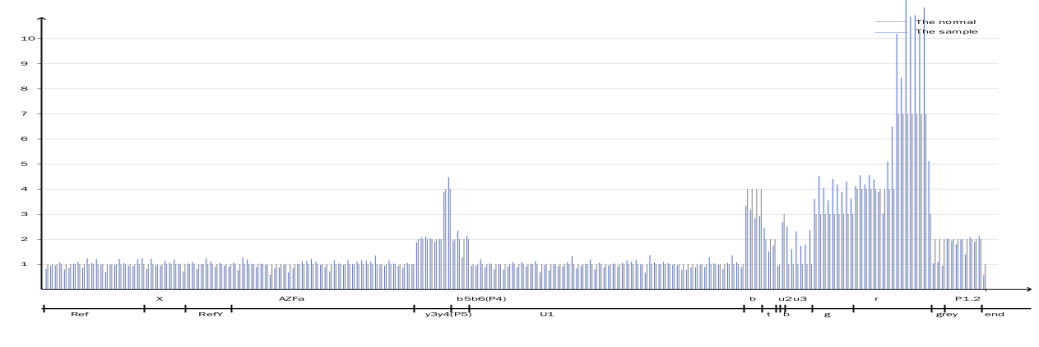

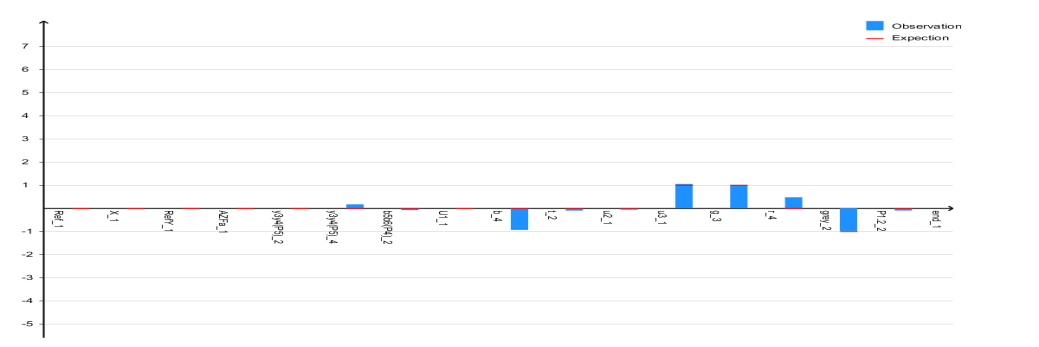


**J: gr/gr del,b2/b4 dup**

**K: gr/gr del,b2/b4 dup twice**


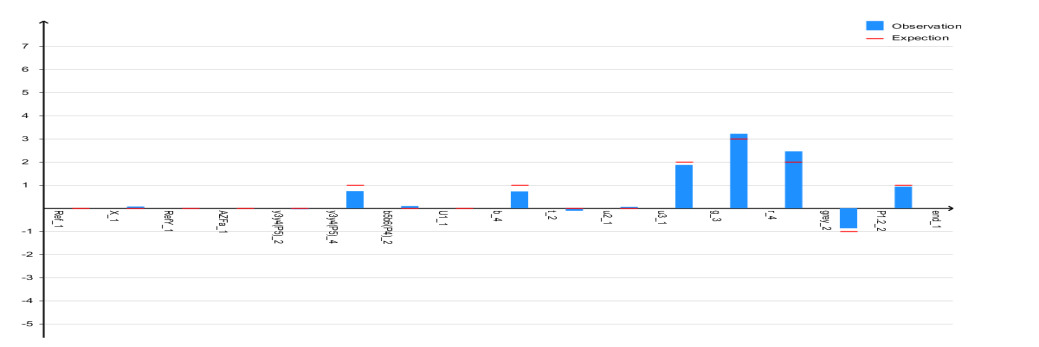

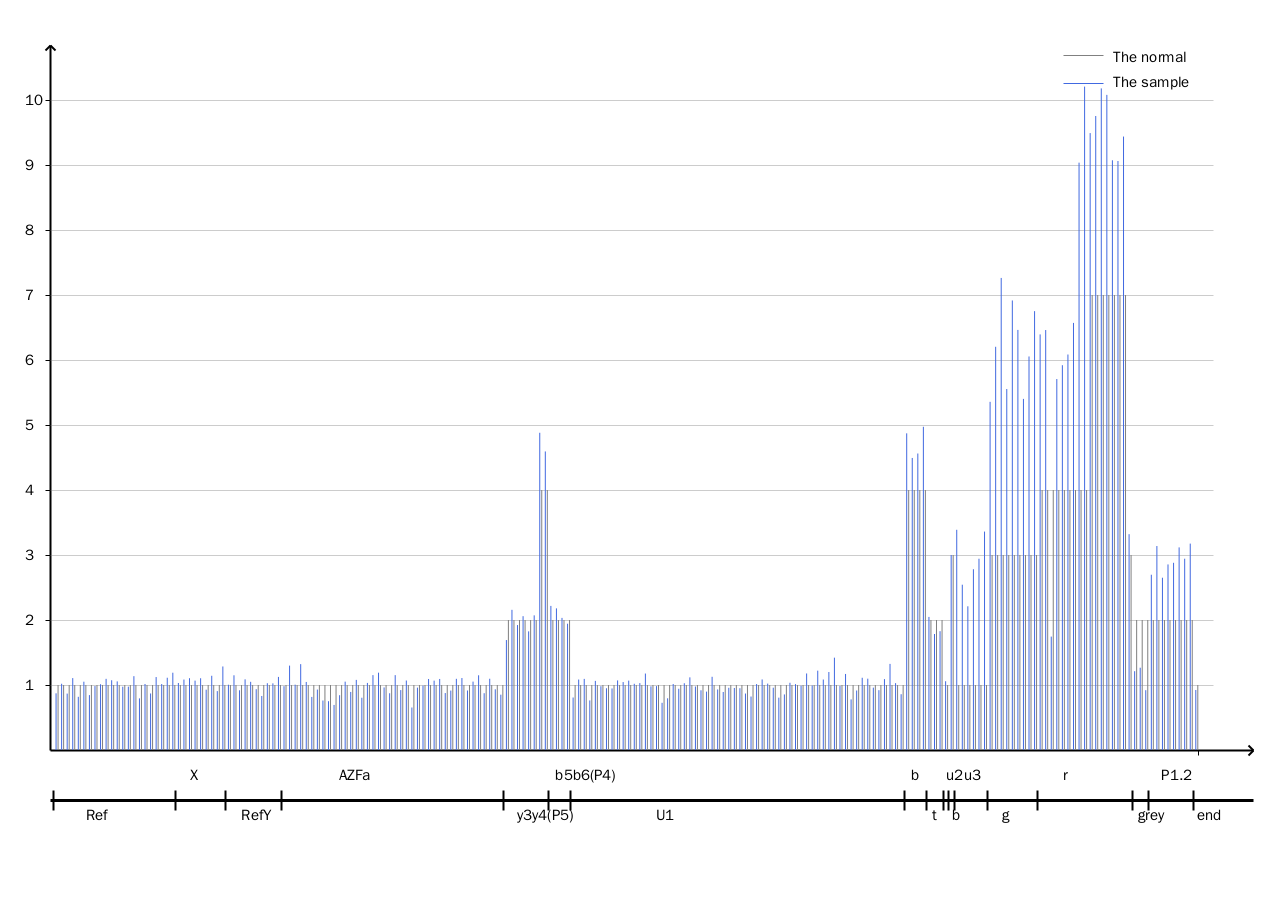


**L: gr/gr dup**


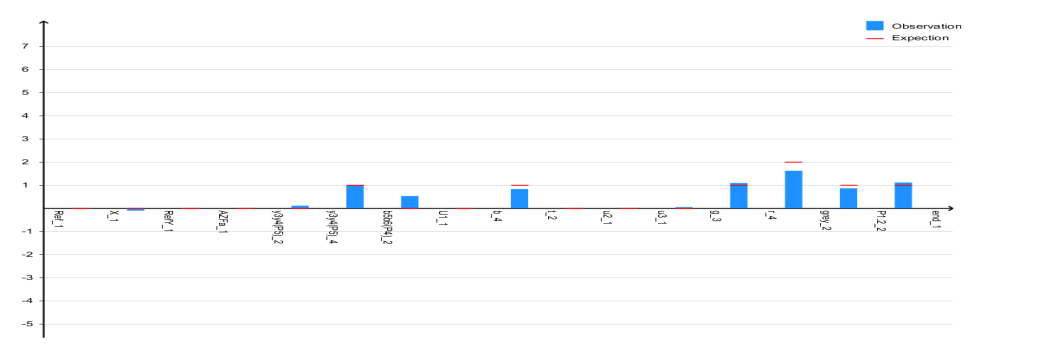

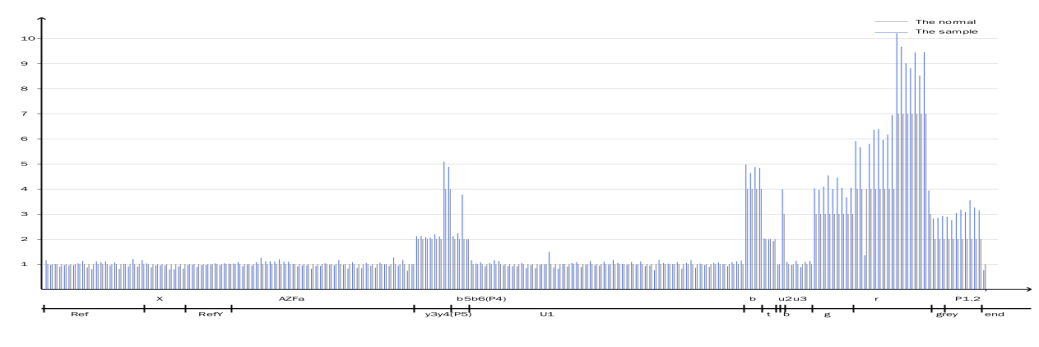


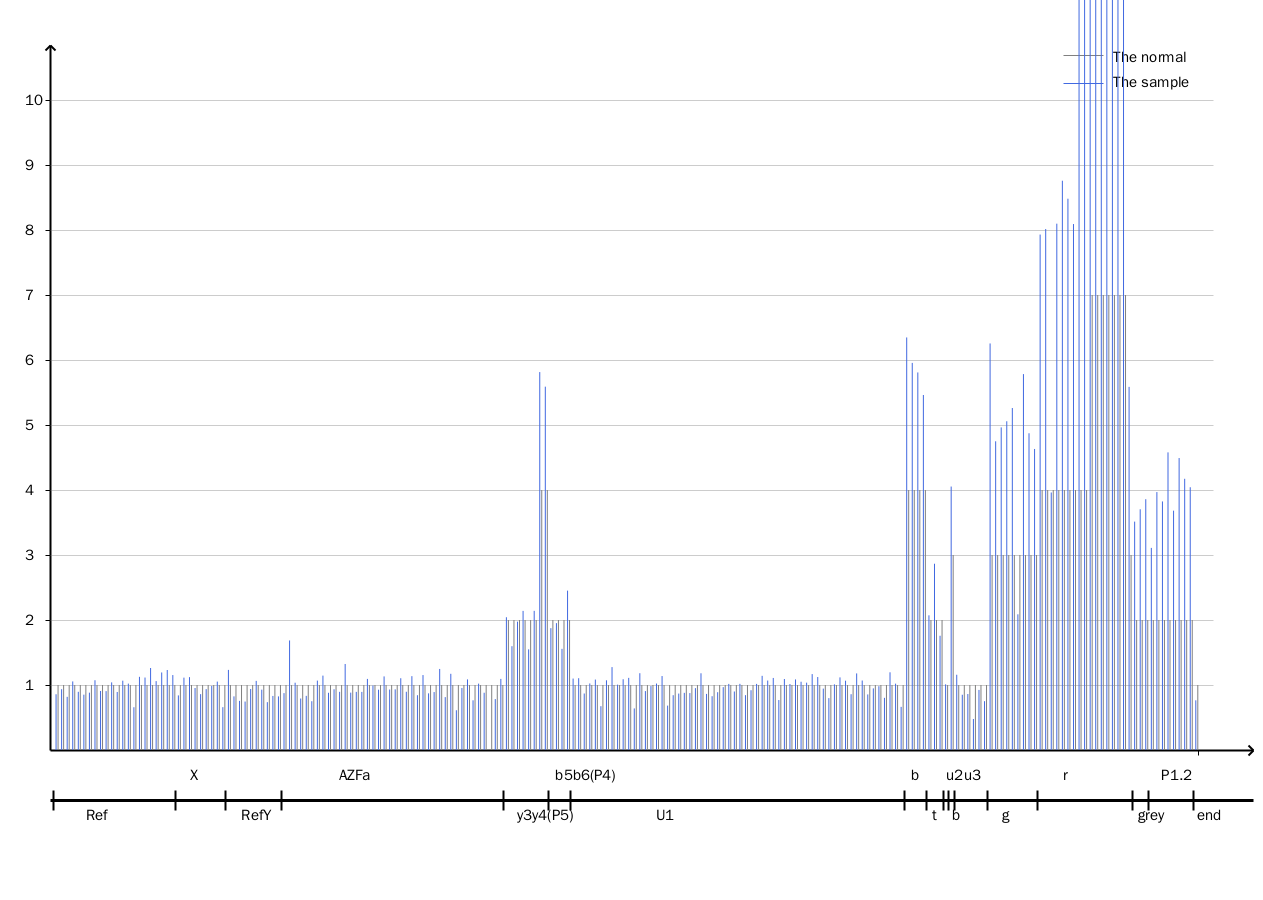

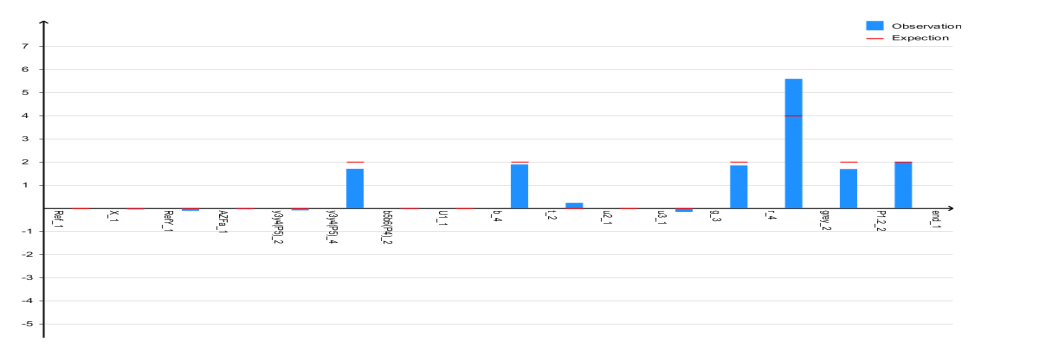


**M: gr/gr dup twice**

**N: b2/b3 dup**


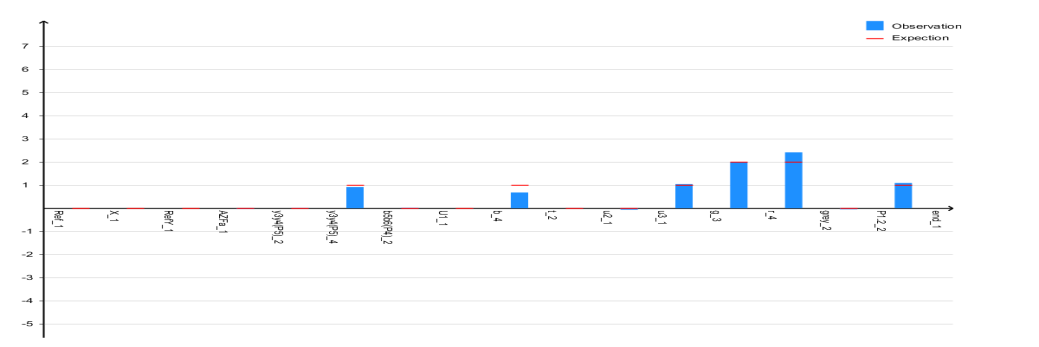

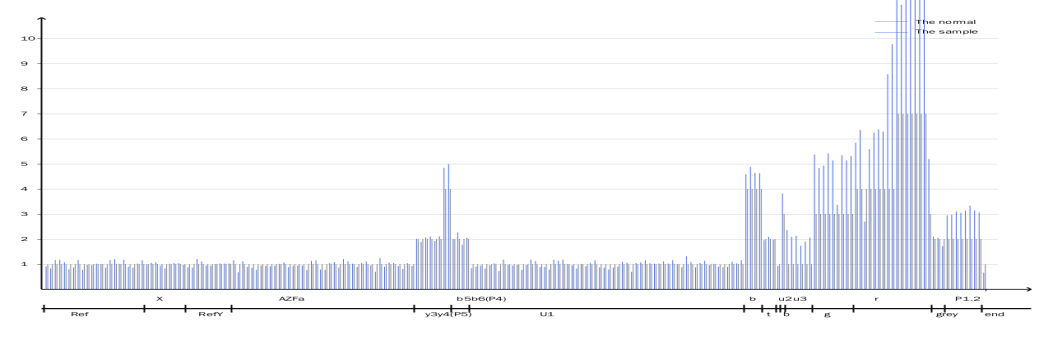


**O: b2/b4 dup**


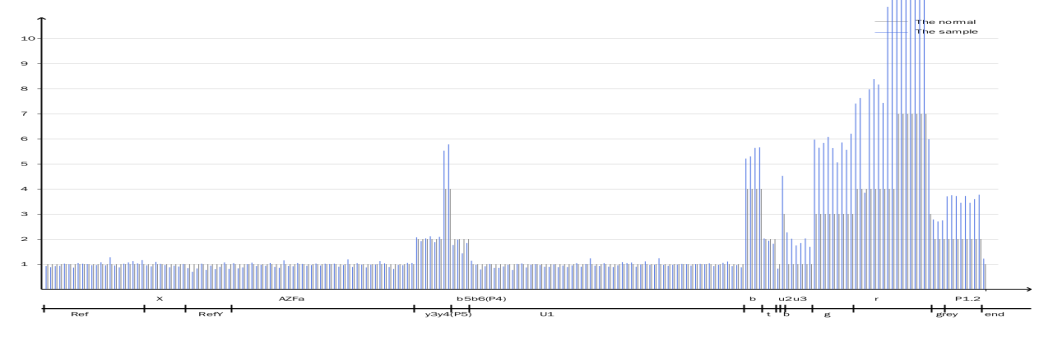

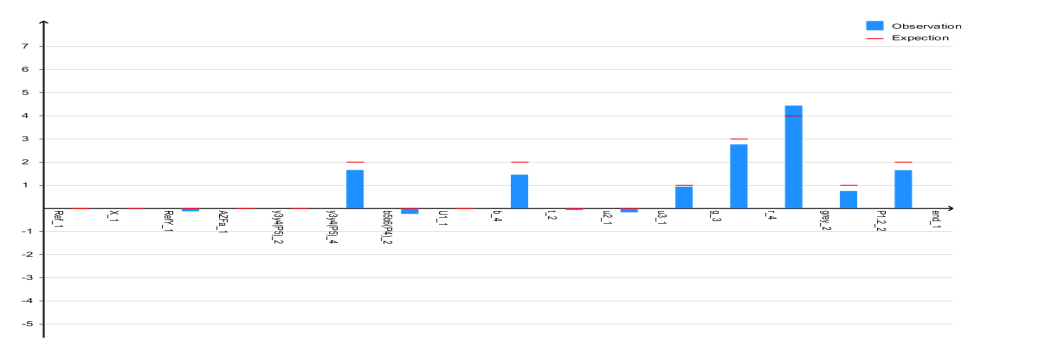


**P: b1/b3 dup**


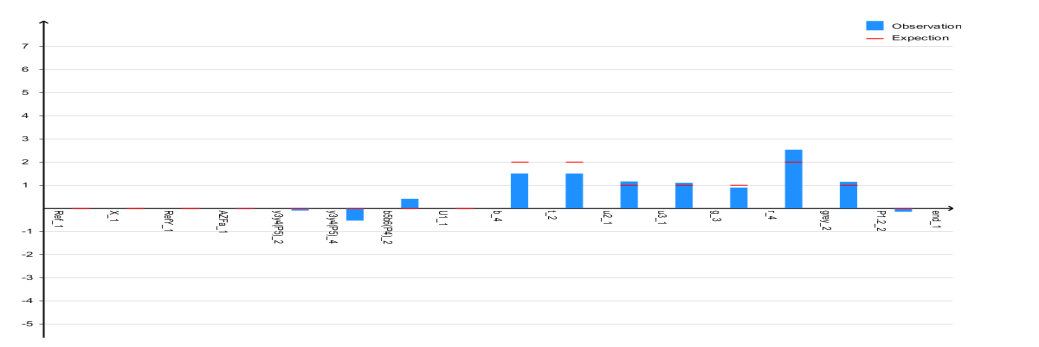

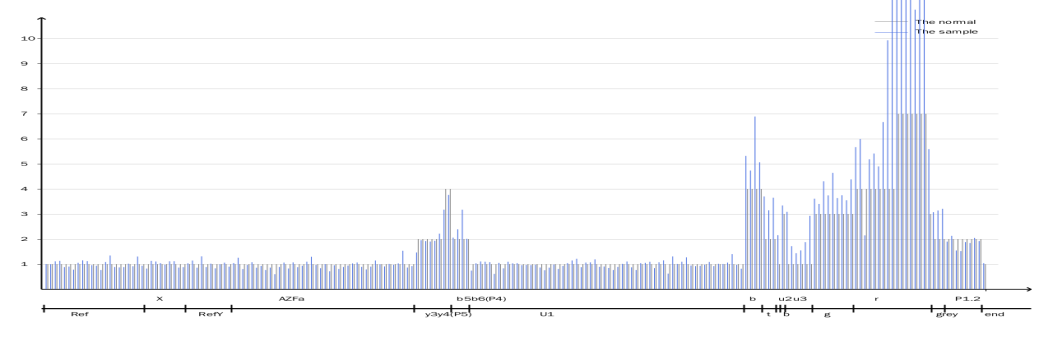


**Q: t1/t2 dup**


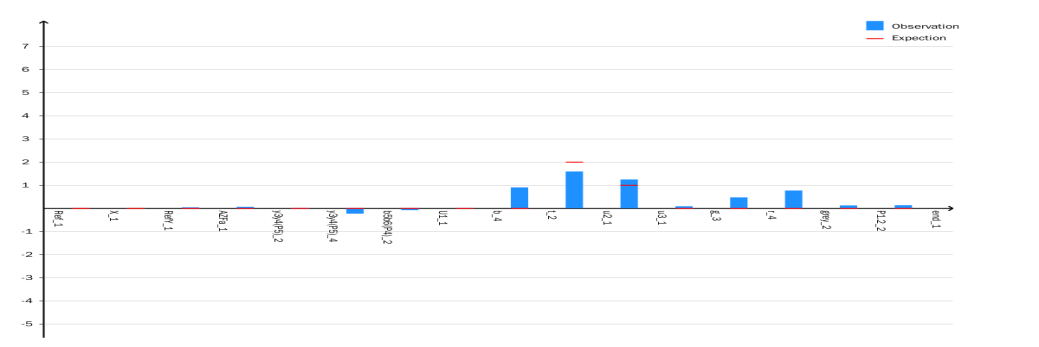

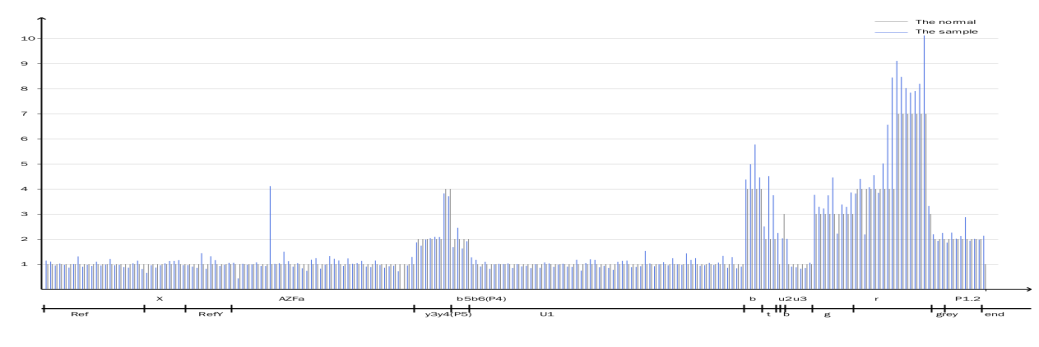


Supplementary Figure S6 Targeted NGS-based detection of Y chromosome microdeletions and duplications in the AZF region. This comprehensive figure illustrates 17 types of distinct CNV (A-Q) in the Azoospermia Factor (AZF) region, Each type of CNV is represented by two plots: a simplified CNV plot (left) and a detailed probe signal intensity plot (right).

**1.2 Supplementary tables**

**Supplementary Table S1 Primers for qPCR**

| **Primer** | **Forward Sequence (5’-3’)** | **Reverse Sequence (5’-3’)** |
| --- | --- | --- |
| ZFX/Y | ACC RCT GTA CTG ACT GTG ATT ACA C | GCA CYT CTT TGG TAT CYG AGA AAG T |
| sY254 | GGG TGT TAC CAG AAG GCA AA | GAA CCG TAT CTA CCA AAG CAG C |
| sY1291 | TAA AAG GCA GAA CTG CCA GG | GGG AGA AAA GTT CTG CAA CG |
| sY1191 | CCA GAC GTT CTA CCC TTT CG | GAG CCG AGA TCC AGT TAC CA |
| sY242 | ACACAGTAGCAGCGGGAGTT | TCTGCCACTAAACTGTAAGCTCC |
| sY802 | ATCACAGTGGGGCAAGTTTC | TGGAGGTGTCTCAGTGGTGA |

**Supplementary Table S2 **qPCR validation site selection and expected results for 17 types of AZF deletion and duplication****

| **Serial No.** | **Type** | **Mode** | **qPCR Validation site** | **Expected results** |
| --- | --- | --- | --- | --- |
| A | b2/b4 del | b2/b4 del | sY254,sY1291,sY1191 | sY254, sY1291, and sY1191 loci are absent. |
| B | b1/b3 del | b1/b3 del-only | sY254,sY1291,sY1191 | sY1191 and sY1291 loci are absent, while sY254 is present. |
| C |  | b2/b4 dup+b1/b3 del | sY254,sY1291,sY1191,sY242 | sY1191 and sY1291 have a single copy, sY254 has more than 7 copies, and sY242 has 6 copies. |
| D |  | gr/gr dup+b1/b3 del | sY254,sY1291,sY1191,sY242 | sY1191 is absent, sY1291 has a single copy, theoretically sY254 has 7 copies, and sY242 has 4 copies. |
| E | b2/b3 del | b2/b3 del-only | sY254,sY1291,sY1191,sY242 | sY1191 is absent, sY1291 has a single copy, theoretically sY254 has 4 copies, and sY242 has 2 copies. |
| F |  | b2/b3 del+b3/b4 dup | sY254,sY1291,sY1191,sY242 | sY1191 is absent, sY1291 has 2 copies, sY254 is present, and sY242 has 4 copies. |
| G |  | b2/b3 del+grey1,r1,r2 dup | sY254,sY1291,sY1191,sY242 | sY1191 is absent, sY1291 is suspected to have 2 copies, sY254 is present, and sY242 has 4 copies. |
| H |  | b2/b3 del+grey1,r2 dup | sY254,sY1291,sY1191,sY242 | sY1191 is absent, sY1291 is suspected to have 2 copies, sY254 is present, and sY242 has 3 copies. |
| I | gr/gr del | gr/gr del-only | sY254,sY1291,sY1191 | sY1191 has a single copy, sY1291 is absent, and sY254 is present. |
| J |  | gr/gr del+b2/b4 dup | sY254,sY1291,sY1191 | sY1191 has 2 copies, sY1291 is absent, and sY254 is present. |
| K |  | gr/gr del+b2/b4 dup twice | sY254,sY1291,sY1191 | sY1191 has 3 copies, sY1291 is absent, and sY254 is present. |
| L | dup only | gr/gr dup | sY254,sY1291,sY1191 | sY1191 has a single copy, sY1291 has 2 copies, and sY254 is present. |
| M |  | gr/gr dup twice | sY254,sY1291,sY1191 | sY1191 has a single copy, sY1291 has 3 copies, and sY254 is present. |
| N |  | b2/b3 dup | sY254,sY1291,sY1191 | sY1191 has 2 copies, sY1291 has a single copy, and sY254 is present. |
| O |  | b2/b4 dup | sY254,sY1291,sY1191,sY242 | Each of sY1191 and sY1291 has 2 copies, sY254 is present, and sY242 has 8 copies. |
| P |  | b1/b3 dup | sY254,sY1291,sY1191,sY242 | Each of sY1191 and sY1291 has 2 copies, sY254 is present, and sY242 has 6 copies. |
| Q |  | t1/t2 dup | sY802,sY254, | sY802 has more than 2 copies, and sY254 is present. |

**Note:** Serial No. is a unique identifier for each type of CNVs such as gene deletions or duplications. The type and mode represent different CNVs in the AZFc region, including deletions **(del) and duplications (dup). qPCR validation sites refer to selected specific validation sites for identifying different types of CNVs****. The expected results refer to the detailed description of anticipated qPCR validation outcomes for each type of CNVs, including the presence, absence, or changes in number of validated STS markers.**

**Supplementary Table S3 Comparison of clinical characteristics between AZFc duplication groups and the non-AZF deletion group**

| Characteristic | Non-AZF deletion (n=575) | gr/gr duplication (n=32) | b2/b3 duplication (n=17) |
| --- | --- | --- | --- |
| Female age (y) | 29.66 ± 2.85 | 29.66 ± 3.28 | 28.82 ± 2.77 |
| Female BMI (kg/m2) | 22.92 ± 3.62 | 23.66 ± 3.86 | 25.48 ± 4.46** |
| Duration of infertility (y) | 3.00 (2.00-5.00) | 3.50 (2.00-5.00) | 4.06 ± 2.68 |
| Female basal LH (mU/L) | 4.90 (3.61-6.70) | 4.61 (3.45-5.67) | 5.04 (3.11-6.89) |
| Female AMH (μg/L) | 4.77 (2.98-7.32) | 4.18 (2.96-5.77) | 5.23 (3.09-10.49) |
| Basal antral follicle count | 19.15 ± 5.80 | 21.16 ± 3.67 | 20.71 ± 6.72 |
| Male age (y) | 31.65 ± 4.13 | 32.34 ± 5.61 | 30.94 ± 3.45 |
| Male BMI (kg/m2) | 25.53 ± 4.05 | 26.15 ± 4.28 | 26.85 ± 5.44 |
| Testicular volume (mL) | 11.81 ± 4.07 | 11.40 ± 3.55 | 12.88 ± 3.61 |
| Cause of infertility, n (%) | | | |
| Male factor | 418 (72.70%) | 24 (75.00%) | 13 (76.47%) |
| Tubal | 26 (4.52%) | 2 (6.25%) | 1 (5.88%) |
| Endometriosis | 2 (0.35%) | / | / |
| Anovulatory | 4(0.70%) | / | / |
| Unexplained | 12 (2.09%) | / | 1 (5.88%) |
| Mixed factors | 105 (18.26%) | 6 (18.75%) | 2 (11.76% |
| Other causes | 8 (1.39%) | / | / |
| Seminal volume (ml) | 3.42 ± 1.82 | 3.32 ± 1.59 | 3.92 ± 1.96 |
| Seminal concentration (10^6^/ml) | 5.75 (0.00-26.73) | 6.85 (1.65-37.68) | 26.30 (0.70-53.80)* |
| No.of men with azoospermia, n (%) | 151 (26.31%) | 2 (6.25%) | 3 (17.65%) |
| No.of men with severe oligozoospermia, n (%) | 129 (22.47%) | 9 (28.12%) | 2 (11.76%) |
| No.of men with mild oligozoospermia, n (%) | 91 (15.85%) | 7 (21.88%) | 2 (11.76%) |
| No.of men with normal sperm concentration, n (%) | 203 (35.37%) | 14 (43.75%) | 10 (58.82%) |
| No.of transferred cycles | 1.42 ± 0.73 | 1.56 ± 0.72 | 1.24 ± 0.44 |
| No. of embryos transferred | 2.28 ± 1.26 | 2.66 ± 1.33 | 2.00 ± 0.79 |
| Semen type, n (%) | | | |
| Fresh, n (%) | 485 (84.35%) | 30 (93.75%) | 15 (88.24%) |
| Frozen, n (%) | 90 (15.65%) | 2 (6.25%) | 2 (11.76%) |
| Sperm extraction method, n (%) | | | |
| Ejaculated, n (%) | 456 (79.30%) | 28 (87.50%) | 15 (88.24%) |
| Testicular sperm, n (%) | 119 (20.70%) | 4 (12.50%) | 2 (11.76%) |

Note: Non-AZF deletion group serves as the reference.

Data are presented as mean ± SD for normally distributed variables, median (IQR) for non-normally distributed variables, or n (%) for categorical variable.

Asterisks (*) indicate significant differences compared to the Non-AZF deletion group. Significance levels: *P ≤ 0.05, **P ≤ 0.01, ***P ≤ 0.001.

**Supplementary Table S4 Embryologic and cumulative reproductive outcomes of male patients with or without AZFc duplication after undergoing ICSI treatment**

| Indicators | Non-AZF deletion (n=575) | gr/gr dup(n=32) | b2/b3 dup(n=17) |
| --- | --- | --- | --- |
| Fertilization rate per oocytes retrieval,% (n) | 79.89%(5459/6833) | 78.36%(315/402) | 74.17%(112/151)* |
| 2PN fertilization rate, % (n) | 74.80%(5111/6833) | 74.63%(300/402) | 72.85%(110/151) |
| 2PN cleavage rate,% (n) | 98.40%(5029/5111) | 97.67%(293/300) | 99.09%(109/110) |
| High-quality embryo rate,% (n) | 52.53%(2685/5111) | 52.33%(157/300) | 50.91%(56/110) |
| Blastocyst formation rate,% (n) | 62.24%(2065/3318) | 58.91%(119/202) | 66.67%(46/69) |
| implantation rate,% (n) | 51.91%(680/1310) | 43.53%(37/85) | 58.82%(20/34) |
| Biochemical pregnancy rate per transfer, % (n) | 72.48%(590/814) | 64.00%(32/50) | 80.95%(17/21) |
| Clinical pregnancy rate per transfer, % (n) | 65.97%(537/814) | 56.00%(28/50) | 71.43%(15/21) |
| Ongoing pregnancy rate per transfer, % (n) | 58.11%(473/814) | 50.00%(25/50) | 57.14%(12/21) |
| Miscarriage rate per pregnancy, % (n) | 13.04%(70/537) | 10.71%(3/28) | 20.00%(3/15) |
| Live birth rate per transfer, % (n) | 57.00%(464/814) | 50.00%(25/50) | 57.14%(12/21) |
| Biochemical pregnancy rate per woman, % (n) | 90.43%(520/575) | 90.63%(29/32) | 88.24%(15/17) |
| Clinical pregnancy rate per woman, % (n) | 86.78%(499/575) | 84.38%(27/32) | 82.35%(14/17) |
| Ongoing pregnancy rate per woman, % (n) | 80.35%(462/575) | 78.13%(25/32) | 70.59%(12/17) |
| Live birth rate per woman, % (n) | 80.00%(460/575) | 78.13%(25/32) | 70.59%(12/17) |
| No embryo suitable for transfer cycles rate, % (n) | 0.52%(3/575) | 0.00%(0/32)*** | 0.00%(0/17)*** |

Note: Non-AZF deletion group serves as the reference.

Data are presented as % (n/N) for all variables.

Asterisks (*) indicate significant differences compared to the Non-AZF deletion group. Significance levels: *P ≤ 0.05, **P ≤ 0.01, ***P ≤ 0.001.

**Supplementary Table S5 Neonatal outcomes of male patients with or without AZFc duplication after undergoing ICSI treatment**

| Overall outcome | Non-AZF deletion(n=575) | gr/gr dup(n=32) | b2/b3 dup(n=17) |
| --- | --- | --- | --- |
| Singleton live birth delivery rate, % (n) | 76.09%(350/460) | 68.00%(17/25) | 58.33%(7/12) |
| Twin live birth delivery rate, % (n) | 23.91%(110/460) | 32.00%(8/25) | 41.67%(5/12) |
| Birth weight (g), mean±SD | 3049.4 ± 650.2 | 2835.0 ± 781.5 | 2680.0 ± 897.3 |
| Low birth weight rate,< 2500g,% (n) | 17.89%(102/570) | 42.42%(14/33)*** | 41.18%(7/17)** |
| Birth height (cm) | 48.9 ± 3.1 | 47.3 ± 4.1** | 46.6 ± 6.5** |
| **Singleton live birth delivery** | | | |
| Birth weight (g), mean±SD | 3338.5 ± 558.1 | 3277.9 ± 767.1 | 3277.1 ± 697.3 |
| Low birth weight rate, < 2500g, % (n) | 5.43%(19/350) | 11.76%(2/17)* | 14.29%(1/7) |
| Birth height (cm) | 49.9 ± 3.0 | 49.4 ± 3.6 | 50.0 ± 1.6 |
| **Twin live birth delivery** | | | |
| Birth weight (g), mean±SD | 2589.5 ± 506.0 | 2364.4 ± 465.4 | 2262.0 ± 797.3 |
| Low birth weight rate, < 2500g, % (n) | 37.73%(83/220) | 75.00%(12/16)** | 60.00%(6/10) |
| Birth height (cm) | 47.2 ± 2.7 | 45.1 ± 3.4** | 44.3 ± 7.6** |

Note: Non-AZF deletion group serves as the reference.

Data are presented as % (n/N) for categorical variables and mean ± SD for continuous variables.

Asterisks (*) indicate significant differences compared to the Non-AZF deletion group: *P ≤ 0.05, **P ≤ 0.01, ***P ≤ 0.001.

Low birth weight infant (LBW) is defined as birth weight less than 2500g.

**Supplementary Table S6** **Multivariate logistic regression analyses of low birth weight rate in male patients with or without AZFc duplication after undergoing ICSI treatment**

| Indicators | gr/gr dup | b2/b3 dup |
| --- | --- | --- |
| Low birth weight rate, < 2500g, % (n) | | |
| Unadjusted OR (95% CI) | 3.38 (1.64, 6.97) *** | 3.21 (1.19, 8.64) * |
| Adjusted OR (95% CI) | 7.91 (2.66, 23.55) *** | 5.59 (1.46, 21.50) * |
| Singleton live birth delivery: low birth weight rate, < 2500g, % (n) | | |
| Unadjusted OR (95% CI) | 2.32 (0.49, 10.90) | 2.90 (0.33, 25.35) |
| Adjusted OR (95% CI) | 9.87 (0.19, 504.88) | 29.61 (0.90, 973.98) |
| Twin live birth delivery: low birth weight rate, < 2500g, % (n) | | |
| Unadjusted OR (95% CI) | 4.95 (1.55, 15.86) ** | 2.48 (0.68, 9.03) |
| Adjusted OR (95% CI) | 8.78 (2.20, 35.00) ** | 4.77 (1.03, 22.09) * |

Notes: OR, Odds Ratio; CI, Confidence Interval; Non-AZF deletion group serves as the reference.

Asterisks (*) indicate significant differences: *P ≤ 0.05, **P ≤ 0.01, ***P ≤ 0.001.

Results are presented as OR with a 95% CI for both unadjusted and adjusted models.

Analyses were performed separately for overall outcomes, singleton live births, and twin live births.

The adjusted model includes the following covariates: female age, duration of infertility, gestational age at delivery, total number of fetuses, female body mass index (BMI), male age, seminal concentration, semen type, sperm extraction method, pregnancy complications (e.g., gestational diabetes, gestational hypertension), Fresh or frozen embryo transfer, number of transfer cycle. Low birth weight infant (LBW) is defined as birth weight less than 2500g.

**Supplementary Table S7 Multivariate logistic regression analyses of embryologic and cumulative reproductive outcomes from one complete ICSI treatment cycle in male patients with or without AZFc duplication**

| Indicators | gr/gr dup | b2/b3 dup |
| --- | --- | --- |
| **Fertilization rate per oocytes retrieval** | | |
| Unadjusted OR (95% CI) | 0.90 (0.59, 1.37) | 0.72 (0.30, 1.75) |
| Adjusted OR (95% CI) | 0.89 (0.58, 1.37) | 0.82 (0.36, 1.84) |
| **2PN fertilization rate** | | |
| Unadjusted OR (95% CI) | 0.98 (0.66, 1.47) | 0.90 (0.39, 2.10) |
| Adjusted OR (95% CI) | 0.89 (0.60, 1.32) | 0.95 (0.44, 2.05) |
| **2PN cleavage rate** | | |
| Unadjusted OR (95% CI) | 0.70 (0.28, 1.76) | 1.82 (0.27, 12.30) |
| Adjusted OR (95% CI) | 0.65 (0.26, 1.60) | 2.57 (0.36, 18.19) |
| **High-quality embryo rate** | | |
| Unadjusted OR (95% CI) | 0.99 (0.71, 1.40) | 0.94 (0.51, 1.72) |
| Adjusted OR (95% CI) | 0.99 (0.69, 1.42) | 0.99 (0.55, 1.79) |
| **Blastocyst formation rate** | | |
| Unadjusted OR (95% CI) | 0.87 (0.64, 1.19) | 1.16 (0.44, 3.05) |
| Adjusted OR (95% CI) | 0.83 (0.57, 1.21) | 1.29 (0.52, 3.22) |
| **implantation rate** | | |
| Unadjusted OR (95% CI) | 0.6 (0.4, 1.0) | 1.3 (0.7, 2.5) |
| Adjusted OR (95% CI) | 0.69 (0.50, 0.97)* | 1.07 (0.59, 1.97) |
| **Biochemical pregnancy rate per transfer** | | |
| Unadjusted OR (95% CI) | 0.68 (0.36, 1.30) | 1.63 (0.59, 4.51) |
| Adjusted OR (95% CI) | 0.73 (0.42, 1.27) | 1.73 (0.60, 5.04) |
| **Clinical pregnancy rate per transfer** | | |
| Unadjusted OR (95% CI) | 0.66 (0.37, 1.18) | 1.30 (0.56, 2.99) |
| Adjusted OR (95% CI) | 0.62 (0.37, 1.04) | 1.17 (0.53, 2.61) |
| **Ongoing pregnancy rate per transfer** | | |
| Unadjusted OR (95% CI) | 0.72 (0.41, 1.28) | 0.96 (0.48, 1.93) |
| Adjusted OR (95% CI) | 0.82 (0.49, 1.35) | 0.86 (0.39, 1.87) |
| **Miscarriage rate per pregnancy** | | |
| Unadjusted OR (95% CI) | 0.99 (0.51, 1.93) | 1.49 (0.69, 3.18) |
| Adjusted OR (95% CI) | 0.82 (0.38, 1.79) | 1.29 (0.55, 3.02) |
| **Live birth rate per transfer** | | |
| Unadjusted OR (95% CI) | 0.76 (0.43, 1.34) | 1.01 (0.50, 2.02) |
| Adjusted OR (95% CI) | 0.90 (0.54, 1.49) | 0.90 (0.40, 2.02) |
| **Biochemical pregnancy rate per woman** | | |
| Unadjusted OR (95% CI) | 1.02 (0.30, 3.47) | 0.79 (0.18, 3.56) |
| Adjusted OR (95% CI) | 1.40 (0.29, 6.74) | 1.58 (0.30, 8.28) |
| **Clinical pregnancy rate per woman** | | |
| Unadjusted OR (95% CI) | 0.82 (0.31, 2.20) | 0.71 (0.20, 2.53) |
| Adjusted OR (95% CI) | 0.82 (0.25, 2.62) | 1.06 (0.27, 4.20) |
| **Ongoing pregnancy rate per woman** | | |
| Unadjusted OR (95% CI) | 0.87 (0.37, 2.07) | 0.59 (0.20, 1.70) |
| Adjusted OR (95% CI) | 0.90 (0.33, 2.47) | 0.93 (0.28, 3.04) |
| **Live birth rate per woman** | | |
| Unadjusted OR (95% CI) | 0.89 (0.38, 2.12) | 0.60 (0.21, 1.74) |
| Adjusted OR (95% CI) | 0.96 (0.35, 2.65) | 0.96 (0.29, 3.19) |

Notes: OR, Odds Ratio; CI:,Confidence Interval; Non-AZF deletion group serves as the reference.

Asterisks (*) indicate significant differences: *P ≤ 0.05, **P ≤ 0.01, ***P ≤ 0.001.

Results are presented as OR with a 95% CI for both unadjusted and adjusted models.

Adjusted model includes the following covariates: female age, female BMI, duration of infertility,female AMH, basal antral follicle count, metaphase II oocytes, male age, male BMI, seminal concentration, semen type, sperm extraction method, number of transferred cycles and number of embryos transferred.
